# The Personalised Randomized Controlled Trial: Evaluation of a new trial design

**DOI:** 10.1002/sim.9663

**Published:** 2023-02-02

**Authors:** Kim May Lee, Rebecca M. Turner, Guy E. Thwaites, A. Sarah Walker, Ian R. White

**Affiliations:** ^1^ Institute of Psychiatry, King's College London London UK; ^2^ MRC Clinical Trials Unit at UCL London UK; ^3^ Centre for Tropical Medicine and Global Health, Nuffield Department of Medicine University of Oxford Oxford UK; ^4^ Oxford University Clinical Research Unit Ho Chi Minh City Vietnam

**Keywords:** indirect evidence, network meta‐analysis, personalised randomization, subgroup analysis

## Abstract

In some clinical scenarios, for example, severe sepsis caused by extensively drug resistant bacteria, there is uncertainty between many common treatments, but a conventional multiarm randomized trial is not possible because individual participants may not be eligible to receive certain treatments. The Personalised Randomized Controlled Trial design allows each participant to be randomized between a “personalised randomization list” of treatments that are suitable for them. The primary aim is to produce treatment rankings that can guide choice of treatment, rather than focusing on the estimates of relative treatment effects. Here we use simulation to assess several novel analysis approaches for this innovative trial design. One of the approaches is like a network meta‐analysis, where participants with the same personalised randomization list are like a trial, and both direct and indirect evidence are used. We evaluate this proposed analysis and compare it with analyses making less use of indirect evidence. We also propose new performance measures including the expected improvement in outcome if the trial's rankings are used to inform future treatment rather than random choice. We conclude that analysis of a personalized randomized controlled trial can be performed by pooling data from different types of participants and is robust to moderate subgroup‐by‐intervention interactions based on the parameters of our simulation. The proposed approach performs well with respect to estimation bias and coverage. It provides an overall treatment ranking list with reasonable precision, and is likely to improve outcome on average if used to determine intervention policies and guide individual clinical decisions.

## 1 INTRODUCTION

In some clinical scenarios, a number of treatment options are available, but particular treatments are contra‐indicated (ie, unsuitable) for particular patients. To address this problem, the challenge is to design a randomized controlled trial (RCT) that can inform treatment choices for all patients. However, two‐arm trials may be inefficient and fail to address the clinical problem, while a standard multiarm trial may exclude patients with any contra‐indication. Both assume a “standard of care” comparator which may not exist.

For example, there is substantial clinical uncertainty over how best to treat multidrug‐resistant infections and a clear need for RCTs to provide robust comparisons of the treatment options available. In the area of carbapenem‐resistant bacterial infections, treatment choices are limited and include high‐dose carbapenems, older, potentially toxic drugs, newer drugs and combinations of drugs, and there is no consensus over which regimens should be used and no obvious “standard of care” control.^1^, ^2^, ^3^ Despite the urgency of the clinical questions, few randomized trials have been carried out to compare the effectiveness of different treatment regimens for patients with carbapenem‐resistant bacterial infections.^4^ The majority of studies that have been performed have been small regulatory studies of new antibiotics compared to a wide variety of “standard of care” regimens, that do not inform the key public health questions. The requirement that recruited patients must be eligible for randomization between all treatments included in the trial is problematic, because many patients with carbapenem‐resistant infections have one or multiple contraindications. Causes of contraindications include the antimicrobial susceptibility of the infecting organism to other antibiotics (which is highly variable) or the patient's medical history, allergies or conditions such as impaired renal function. These issues make it difficult to find any two specific regimens to which large numbers of patients with multidrug‐resistant infections can be randomized, and cause severe recruitment difficulties in RCTs.

When treating a patient with a multidrug‐resistant infection, the clinician wishes to know which of the regimens that the patient could reasonably take will provide the greatest probability of success. This scenario requires an analysis that is analogous to network meta‐analysis,^5^ in which multiple treatments are compared in an evidence synthesis and the aim is to rank treatments and provide recommendations about the best treatments. We previously proposed a new trial design that borrows from network meta‐analysis methodology, called a “Personalised Randomized Controlled Trial” (PRACTical) design.^4^ For a network of treatments of interest, each patient would be randomized only between regimens for which they are eligible, with equal allocation probability within the sets of eligible regimens: we call this personalised randomization list their treatment “pattern”. Note that this “personalization” is related to the treatments that the patients are eligible for, which is a different notion to the idea of precision/stratified medicine where some patients are expected to respond differently to a given treatment.

It is not new to randomize only between certain trial arms: for example, the RECOVERY trial of COVID‐19 treatments^6^ randomized any patient who was eligible for control and at least one experimental treatment, while in the CAST trial of cardiac arrhythmia treatments^7^ randomization lists differed according to the patient's cardiac ejection fraction measurement. However, these trials aimed to compare experimental treatments with control directly using standard analysis methods. The PRACTical design^4^ differs in that it aims to produce an overall ranking of the treatments that can be used in conjunction with a personalised list of eligible treatments to suggest a preferable treatment for each patient; it need not have a control treatment; and it includes any patient who is eligible for at least two of the treatments.

In this article, we focus on the analysis of the PRACTical design that has been proposed by Walker et al,^4^ who have discussed the ideas of the novel design and suggested how it might be analyzed but have not provided a detailed description or thorough investigation of the analysis approach. We evaluate the performance of several possible analysis methods in a simulation study. We propose two methods that make full use of indirect evidence on treatment comparisons, by combining randomized evidence from subgroups of patients who were eligible for different treatment patterns. The first of these is motivated by standard methods for network meta‐analysis. A subgroup of patients with the same pattern forms the unit in analysis of the entire network of treatments, analogous to an individual trial within a network meta‐analysis. Subgroups of patients with different treatment patterns are pooled in a combined analysis. This method is likely to perform poorly if the number of patterns is very large, since one parameter is used for each pattern. A second method avoids this problem by estimating each pairwise comparison separately from the appropriate subset of the data and combining the estimates for different comparisons. Both the above methods assume that indirect evidence is consistent with direct evidence, and we compare them with simpler methods using less indirect evidence on treatment comparisons or using direct evidence only. The aim of the simulation study is to compare methods with respect to bias, precision, and coverage of parameter estimates. We do this first when indirect and direct evidence are consistent and then second when they are inconsistent, in order to understand first the benefits and then the costs of using indirect evidence. We also propose and use new performance measures to evaluate the impact of the rankings on treatment decisions.

The article is structured as follows. First, we present a clinical example that employs a PRACTical design. We are developing methods for future data analysis, and no trial data are available at the time of writing. We then describe our proposed methods for analysis, together with the simpler methods to be used for comparison. Next, we set out the scenarios considered in the simulation study and the performance measures to be evaluated. The results are presented in the following section and we conclude with a discussion of our findings.

## 2 CLINICAL EXAMPLE

Table 1 shows an example of how individual patients might be randomized in a PRACTical design evaluating treatments or regimens for patients with carbapenem‐resistant bacterial infections.^4^ We have assumed that 10 treatments, including single drugs and combinations of drugs, will be compared in the trial–there is no “standard of care”. Each patient would be randomized only to those treatments that were judged clinically appropriate for the patient. For example, patient 2 in Table 1 is eligible for randomization to any treatment except treatment T9 (with equal allocation probability of 1/9), whereas patient 3 is eligible only for treatments T1, T2, T3, and T5 (with equal allocation probability of 1/4). We have assumed that all 10 treatments are available at each site recruiting patients, that recruiting physicians are willing to allow randomization to any treatment if clinically appropriate (ie, minimal physician bias) and that tests for antimicrobial susceptibility can be completed within an acceptable (<48 hour) period.

**TABLE 1:** Example personalised randomization lists in a PRACTical design evaluating treatments for carbapenem‐resistant infections (4)

Possible regimens	Patient 1 History of moderate–severe cephalosporin allergy	Patient 2 History of myocardial infarction	Patient 3 Meropenem MIC ≥64	Patient 4 VAP/HAP	Patient 5 *Pseudomonas aeruginosa* infection	Patient 6 Known Class B infection
T1: plazomicin						No
T2: ceftazidime/avibactam	No					No
T3: cefiderocol	No					
T4: high‐dose meropenem			No			
T5: polymixin B ± zidovudine				No		
T6: high‐dose meropenem + ertapenem			No		No	
T7: high‐dose meropenem + imipenem			No			
T8: high‐dose meropenem + polymixin B ± zidovudine			No	No		
T9: high‐dose meropenem+high‐dose tigecyline		No	No		No	
T10: high‐dose meropenem + fosfomycin			No			
Physician decides patient can be randomized to	All except T2 and T3	All except T9	T1, T2, T3, T5	All except T5 and T8	All except T6 and T9	All except T1 and T2

*Note*: For each column, the personalised randomization list consists of all the regimens not marked “No”.

Abbreviations: MIC, median inhibitory concentration, VAP/HAP, ventilator‐acquired pneumonia/hospital‐acquired pneumonia.

## 3 ANALYSIS METHODS

### 3.1 Notation

Let the treatments be j=T1,T2,T3,…. We use “pattern” to refer to the set of allowable treatments for a patient and “subgroup” to refer to the set of patients with this pattern. Let a pattern be $S_{k}\subseteq \left\{T1,T2,T3,\ldots \right\}$ and $m_{k}$ be the number of eligible treatments in $S_{k}$, for subgroup k=1,…,K. Let *N* be the total number of patients in the trial, $\lambda_{k}$ the prevalence of pattern $S_{k}$ in the target population and $n_{k}=$ *N* $\lambda_{k}$ the expected sample size of patients who have pattern $S_{k}$. The event probability for patient *i* who receives treatment *j* in subgroup k is $P_{\mathrm{jk}}=P\left(y_{\left(i\right)\mathrm{jk}}=1\right)$ where $y_{\left(i\right)\mathrm{jk}}$ is a binary variable with 1 indicating an event and 0 otherwise. We consider mortality as the event of interest in the illustration.

### 3.2 Approach

We only consider analyses that respect the randomization. That is, we are prepared to assume comparability of participants in the same subgroup randomized to different treatments, but not of participants in different subgroups. This requires controlling for pattern in some, but not all, analyses. We present analyses in increasing order of their use of indirect evidence. Method A is the standard subgroup analysis approach while methods B1, B2, B3, C, and D, are novel approaches. We describe extending the models to include baseline covariates in the discussion.

### 3.3 A: Subgroup‐specific analysis

We first describe an analysis that uses no indirect evidence and instead assumes a different set of treatment effects in each subgroup. This is achieved by fitting a model to each subgroup k separately:

(1) $$\text{logit}\left(P_{\mathrm{jk}}\right)=\alpha_{\left(k\right)}+\psi_{j\left(k\right)},$$

where $\psi_{j\left(k\right)}$ represents the log odds ratio comparing treatment j with treatment $j^{\prime }$ in pattern $S_{k}$, $j^{\prime }$ denotes a reference treatment for pattern $S_{k}$ (typically the first treatment in the randomization list), $\alpha_{\left(k\right)}$ is the log odds for the reference treatment of pattern $S_{k}$ and we constrain $\psi_{j^{\prime }\left(k\right)}=0$. Then a separate ranking is created for each pattern and is unaffected by the choice of $j^{\prime }$. This approach is likely to be very inefficient, especially with small subgroups, since it does not combine evidence across subgroups.

### 3.4 B: Partly pooled analyses

We next describe three analyses that use increasing amounts of indirect evidence. Because they do not use all the indirect evidence, they create a separate ranking for each pattern, like method A. We describe the analysis for subgroup k.

Method B1 uses evidence from subgroup k and any other subgroup l that includes the eligible treatments for subgroup k ($S_{l}⊃S_{k}$). Model (1) is fitted to these data. Optionally, model (1) can be extended to control for pattern $S_{k}$: that is, in this model estimating treatment effects for subgroup k, a dummy variable can be included for each other pattern $S_{l}$. However, controlling for pattern is not essential, since each pattern contributes equally to all treatments in $S_{k}$. Also, patients in patterns other than $S_{k}$ who are randomised to treatments outside $S_{k}$ may be included or excluded in the analysis: this makes little difference, since dummies for their treatments are included in the model. Our analyses controlled for pattern and excluded patients randomized to treatments outside $S_{k}$. More specifically, letting $P_{\mathrm{jl}\left(k\right)}$ be the outcome probability for an individual receiving treatment j in pattern $S_{l}$ in the model for subgroup k, we fit

(2) $$\begin{array}{cc} & \text{logit}\left(P_{\mathrm{jl}\left(k\right)}\right)=\alpha_{l\left(k\right)}+\psi_{j\left(k\right)}, \end{array}$$

where $\psi_{j^{\prime }\left(k\right)}=0$. Here pattern l modifies the $\alpha_{l\left(k\right)}$ term (we control for pattern) but not the $\psi_{j\left(k\right)}$ term (we assume common treatment effects across patterns $S_{l}$, for given pattern $S_{k}$).

Method B2 uses evidence from pattern $S_{k}$ and certain other patterns that compare treatments in pattern $S_{k}$. Specifically, again using a pattern‐specific reference treatment $j^{\prime }\in S_{k}$, we estimate each pairwise comparison between treatments j and $j^{\prime }$, for each $j\in S_{k},j\neq j^{\prime }$. Estimation is done by fitting model (1) to all patterns that include both $j^{\prime }$ and j. As in B1, we choose to adjust for pattern using model (2), and to exclude participants not randomized to $j^{\prime }$ or j. Ranking is then done by comparing the pairwise comparisons. Note that method B2 is sensitive to the choice of pattern‐specific reference treatment.

Method B3 improves on B2 by using evidence from pattern $S_{k}$ and all other patterns that compare any two or more treatments in pattern $S_{k}$: thus it uses all direct evidence about pairwise comparisons between treatments in pattern $S_{k}$, not just the direct evidence that relates to a reference treatment $j^{\prime }$, and so is not sensitive to the choice of $j^{\prime }$. The estimated comparisons are unlikely to be exactly consistent: for example, it is unlikely that the T3‐T1 contrast would equal the sum of the T3‐T2 and T2‐T1 contrasts. To obtain unique contrasts (but still specific to pattern $S_{k}$) we fit model (2) that allows for differences between patterns, which is necessary because pattern is now confounded with treatment. The rankings for pattern $S_{k}$ are derived by comparing the estimates of the coefficients $\psi_{j\left(k\right)}$ for the different treatments j.

### 3.5 C: Network‐based approach

The above methods do not use all the indirect evidence, which is inefficient if indirect evidence is consistent with direct evidence, and they produce a different ranking for each pattern, which makes the results difficult to communicate. We next use all the indirect evidence to produce a single ranking for all patterns. We fit a single model to all the data, adjusting for pattern:

$$\text{logit}\left(P_{\mathrm{jk}}\right)=\alpha_{k}+\psi_{j},$$

where the intercept $\alpha_{k}$ is the log odds for the baseline risk of patients in subgroup k if treated with a reference treatment $j^{\prime }$, and $\psi_{j}$ is the log odds ratio for treatment j compared with treatment $j^{\prime }$, with $\psi_{j^{\prime }}=0$. Including the effect of pattern in the model is again essential to respect the randomization. Rankings are obtained by comparing the estimates $\psi_{j}$.

### 3.6 D: Pairwise trials approach

A disadvantage of method C is that it includes one parameter $\alpha_{k}$ for each pattern, which may be inefficient if we have many patterns; in the extreme case where every participant has a unique pattern, method C would not produce estimates. Method D is like method C in that it uses all the indirect evidence, but it avoids introducing one parameter per pattern. It does this by recognizing that two treatments can be compared by comparing all participants who were randomized to one of the treatments but could have been randomized to the other. The approach for each pairwise comparison is a simple comparison of proportions, with the restriction that we do not adjust for pattern and we do not include treatments other than the two being compared. Unlike method B2, however, method D combines evidence from all pairwise comparisons by the following steps.

1. Create the data set required for each pairwise comparison.
2. Stack all these pairwise data sets on top of one another.
3. Analyze the stacked data using a model with a term for the pairwise data set and a term for treatment.
4. Compute standard errors using a sandwich variance to reflect the dependence of the different records for each individual.

In practice this is done by creating multiple copies of each record, each relating to a different pairwise comparison. For example, consider a participant who is eligible for a treatment set, {T1, T2, T4, T5} and is randomized to T2: we create 3 copies of that participant's record and label them as relating to comparisons T1T2, T2T4, and T2T5. We give all records equal weights, but an alternative would be to use weights equal to the reciprocal of the number of copies. The fitted model can be written as

$$\text{logit}\left(P_{j\left(j^{\prime }\right)}\right)=\alpha_{j\left(j^{\prime }\right)}+\psi_{j}.$$

where $P_{j\left(j^{\prime }\right)}$ is the outcome probability for a record representing an individual randomised to treatment j in the comparison with treatment $j^{\prime }$, and $\alpha_{j\left(j^{\prime }\right)}$ is an intercept representing the comparison. We express the randomization by requiring $\alpha_{j\left(j^{\prime }\right)}=\alpha_{j^{\prime }\left(j\right)}$. Note that pattern is not included in this model ‐ the notation does not refer to k. As for method C, rankings are obtained by comparing the estimates of the $\psi_{j}$.

### 3.7 Strengths and weaknesses

Method C is motivated by network meta‐analysis, which makes the assumptions of joint randomizability, that “a patient could, in principle, be randomized in any of the alternative treatment options”, and transitivity, “that indirect comparison validly estimates the unobserved head‐to‐head comparison”.^8^ These strong assumptions are clearly not true in the PRACTical design, because contra‐indicated treatments are not given, and if given would yield very bad outcomes. Fortunately, the assumptions are stronger than needed for the PRACTical design: we do not need any assumptions about treatments that are contra‐indicated in any particular pattern, because in each pattern we have no data on contra‐indicated treatments, and we draw no conclusions for contra‐indicated treatments.

Instead, the transitivity assumption needs only apply to treatments that are not contra‐indicated. With this restriction, it is the same as the assumption that the treatment effects are the same for all patterns, made by methods C and D. By contrast, the B methods assume the treatment effects are the same for some patterns, and Method A is valid when treatment effects vary across patterns. There is therefore likely to be a bias‐variance trade‐off, with estimates from methods C and D having smaller variance than A and B (because more data are used) but having bias when treatment effects vary across patterns.

Collapsibility of the odds ratio is a further problem^9^: the true odds ratio comparing two treatments is changed when patterns are combined. This means that methods A and C, which estimate pattern‐specific odds ratios, estimate a different estimand from method D, which estimated marginal odds ratios. Methods B estimate pattern‐specific or marginal odds ratios depending on whether they adjust for pattern or not. The impact is likely to be small in practice.

## 4 SIMULATION METHODS

The aim of the simulation study is to compare the methods described above, first when the assumption of no interaction between treatment and pattern holds, and then when it does not.

### 4.1 Description of scenarios/designs

In our simulation study, we consider different trial scenarios/ designs with fixed *N* and $\lambda_{k}$, and varying values for *K*, $\alpha_{k}$, $\psi_{j}$ and sets of patterns $S_{k}$. For each scenario/ design, $S_{k}$ and $n_{k}$ remain the same across all simulated trial replications; patients in each subgroup *k* are randomly assigned to treatment arms according to $S_{k}$ with equal treatment allocation ratios across trial replications, and $y_{\left(i\right)\mathrm{jk}}$ is simulated from a Bernoulli distribution with the corresponding probability $P_{\mathrm{jk}}$.

Table 2 describes the scenarios that we explore in our simulation study. More details are available in the Supplemental Material I Table Appendix S1. For all scenarios, we have N=1000, except for S4 where we have N=2000 (such that method A can produce individual rankings for most subgroups for ease of comparisons). Scenarios S1 to S4 have common treatment effects for all patterns, on the log odds scale. Scenarios S1, S1.1, and S1.2 have K=8 patterns with unequal prevalence rates $\lambda_{k}$ as in the clinical example described above: S1 assumes treatment effects but no pattern effects; S1.1 assumes no treatment or pattern effects; and S1.2 assumes both treatment effects and pattern effects. Note that treatment effects are parameterized in comparison to a baseline risk of 20% so that some are positive and others are negative. The reference treatment in methods C and D is the treatment with the lowest $P_{\mathrm{jk}}$, that is, $P_{\mathrm{jk}}=0.1$, the reference treatment in all other methods is the treatment with the lowest $P_{\mathrm{jk}}$ within a pattern. In addition, we explore settings where there are fewer patterns (ie, K=3 for S2) or more patterns including different numbers $m_{k}$ of treatments (K=20 for S3, S3.1 and S3.2, and K=200 for S4).

**TABLE 2:** Description of scenarios for simulation study

Scenario, S	No. of patterns, *k*	No. of treatment per patterns: min, median, max	Baseline risk	Mortality rate after treatment	Values of $\lambda_{k}$
Without treatment by pattern interactions					
1	8	5, 7, 10	20% ∀k	10% to 30%	−$\lambda_{1}=\lambda_{2}=$ 20%; −$\lambda_{k}=10\%,\;k=3,\ldots ,8$
1.1	Same as S1	Same as S1	Same as S1	20%	Same as S1
1.2	Same as S1	Same as S1	5% to 60%	10% to 72%	Same as S1
2	3	7, 7, 8	Same as S1	Same as S1	1/3
3	20	3, 6, 9	Same as S1	Same as S1	1/20
3.1	20	3, 3, 3	Same as S1	Same as S1	1/20
3.2	20	7, 7, 7	Same as S1	Same as S1	1/20
4	200	3, 5.5, 9	Same as S1	Same as S1	1/200
With treatment by pattern interactions					
5.1	Same as S3	Same as S3	Same as S1	A treatment has the same effect as that in S1 for some patterns but has no impact on patients of other patterns.	1/20
5.2	Same as S3	Same as S3	Same as S1.2	A treatment has the same effect as that in S1.2 for some patterns but has no impact on patients of other patterns.	1/20
6.1	Same as S3	Same as S3	Same as S1	A treatment has the same effect as that in S1 for some patterns but has the opposite effect on patients of other patterns.	1/20
6.2	Same as S3	Same as S3	Same as S1.2	A treatment has the same effect as that in S1.2 for some patterns but has the opposite effect on patients of other patterns.	1/20

*Note*: Ten treatments are considered in all scenarios. More details are available in the Supplemental Material I Table Appendix S1.

The presence of treatment by pattern interactions is considered next. Scenarios S5.1 and S5.2 have an extreme quantitative interaction: each subgroup‐specific treatment effect is either the same as in scenarios 1 and 1.2 respectively, or zero so that they remain at baseline risk. Scenarios S6.1 and S6.2 have qualitative interactions: each subgroup‐specific treatment effect is of the same magnitude as in scenarios 1 and 1.2 but half of them have reversed direction. Note that all patients in S5.1 and S6.1 have the same baseline risk whereas those in S5.2 and S6.2 have baseline risks varying by subgroup.

### 4.2 Estimands

Our first set of estimands is the *treatment contrasts* defined as the pattern‐specific log odds ratios. This is only estimated by methods C and D so is only evaluated there. Our second set of estimands is the *treatment decisions* that will be made for future patients using the results of the trial. This is estimated by all methods so offers a fuller comparison, but it requires development of new performance measures below.

### 4.3 Performance measures

For the treatment contrasts, we use standard performance measures. Let $\Delta_{j}={\hat{\psi }}_{j}-\psi_{j}$ be the differences between the estimated model parameters and the true treatment contrasts. For each treatment contrast, we estimate

- relative bias $E(\Delta_{j}$)/$\;\psi_{j}$,
- mean squared error $E\left(\Delta_{j}^{2}\right)$, and
- coverage probability, P(the 95% confidence interval covers the true parameter value),

where the expectations are taken with respect to the trial replications.

For the treatment decisions, we consider the following measures that evaluate the estimated best treatments for each pattern in terms of the true mortality rates of all eligible treatments in the patterns. Let ${\hat{j}}_{k}=\text{argmi}n_{j\in S_{k}}{\hat{P}}_{\mathrm{jk}}$ denote the estimated best treatment in $S_{k}$, that is, the treatment that has the lowest estimated mortality rate ${\hat{P}}_{\mathrm{jk}}$. This represents the treatment that would be recommended for patients in $S_{k}$ if the simulated trial data were analyzed using a given method. By considering the impact of treating patients with the *estimated* best treatment rather than the truly best treatment or a randomly chosen treatment, we can evaluate the performance of the proposed methods for analysis.

- *Mortality gain*: We first propose to quantify the reduction in mortality if a future population is treated using the results of the trial, compared to being treated using no information. We assume that treatment using no information amounts to a random choice between each patient's eligible treatments, and that the future distribution of subgroups will be the same as in the trial. We therefore define the mortality gain corresponding to the estimated best treatments by
$$E\left[\sum_{k}{\hat{\lambda }}_{k}\left(\mathrm{av}e_{j\in S_{k}}P_{\mathrm{jk}}-P_{{\hat{j}}_{k}k}\right)\right],$$

where ${\hat{\lambda }}_{k}$ is the observed prevalence of $S_{k}$, and the term in round brackets is the mortality gain of the estimated best treatment in $S_{k}$ over the mean (denoted by *ave*) of true mortality rates across all other eligible treatments within $S_{k}$. A high mortality gain is desirable as that implies that the estimated best treatments can reduce the mortality rate considerably.

- *Better treatment probability*: The expected proportion of patients for whom the estimated best treatment leads to an improvement in outcome is defined by
$$E\left[\sum_{k}{\hat{\lambda }}_{k}I\left(P_{{\hat{j}}_{k}k}<\mathrm{av}e_{j\in S_{k}}P_{\mathrm{jk}}\right)\right],$$

where I(.) is an indicator function; the term in the function indicates that the estimated best treatment has a mortality rate less than the average mortality rate in pattern $S_{k}$.

- *Best treatment probability*: The expected proportion of patients for whom the estimated best treatment is truly the best for the corresponding pattern is defined by
$$E\left[\sum_{k}{\hat{\lambda }}_{k}I\left(P_{{\hat{j}}_{k}k}=\min_{j\in S_{k}}P_{\mathrm{jk}}\right)\right].$$

This probability represents how likely it is that each of the estimated best treatments is indeed the most effective treatment, that is, the treatment that leads to the smallest mortality rate in each pattern $S_{k}$.

- *Near‐best treatment probability*: The expected proportion of patients for whom the estimated best treatment is no more than κ% worse than the truly best treatment for the corresponding pattern is defined by
$$E\left[\sum_{k}{\hat{\lambda }}_{k}I\left(P_{{\hat{j}}_{k}k}\leq \min_{j\in S_{k}}P_{\mathrm{jk}}+\kappa \right)\right].$$

This probability represents how likely it is that each estimated best treatment is no more than κ% worse than the most effective treatment in pattern $S_{k}$.

Note that the computation of these performance measures relies on the true value of $P_{\mathrm{jk}}$, that is, the event rate of the outcome variable. More specifically, we assume this event rate is known for all treatments and subgroups; the estimated best treatment ${\hat{j}}_{k}$ is identified from the estimated treatment effects of the analysis model before computing these performance measures using the true rate

### 4.4 Implementation

Except for S4, we performed 50 000 trial replications: this was chosen such that the Monte Carlo simulation error and computational time are within a reasonable range. For S4, we performed 1000 trial replications as methods A, B1, B2, and B3 need to fit a large number of models for this scenario, and computational resources become limited when the trial replications for this particular setting are large. We report the Monte Carlo simulation errors and take these into account when examining the simulation results. When the absolute value of an estimated contrast is greater than 12, for example due to separation,^10^ the corresponding treatments are not considered when estimating the best treatment, and when computing the properties of the estimates from Methods C and D. Moreover, when a method encounters an error in model fitting, for example, nonconvergence, a randomly selected treatment from a pattern is used in the replications. The simulation was done in R version 3.6 using the *stats* and *multiwayvcov* packages, and high‐performance computing facilities. Sample R scripts are available at https://github.com/kimmaylee215/PRACTical and a simulation study plan that was written before the implementation is available in the Supplemental Material II.

## 5 SIMULATION RESULTS

We first compare the properties of the estimated treatment contrasts obtained from methods C and D respectively for S1, S1.1, S1.2, S2, S3, S3.1, S3.2, and S4. We omit the comparisons in the scenarios that have treatment by pattern interactions since the true value of $\psi_{j}$ varies with patterns and therefore the aforementioned properties of the estimates are not meaningful measures in these scenarios.

### 5.1 Properties of estimated treatment contrasts

**FIGURE 1:**
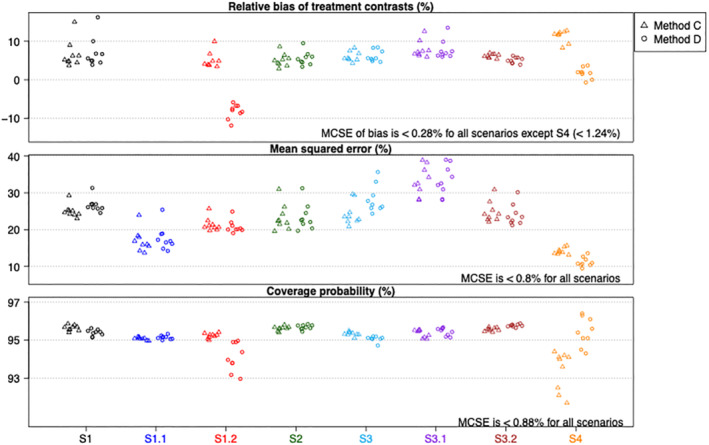
Properties of estimated treatment contrasts for scenarios without treatment by pattern interactions. Numerical values (and Monte Carlo simulation errors) are available in Supplemental Material I Tables S2‐S4 (and Tables S5‐S7)

Figure 1 shows the relative bias, mean squared error and coverage probability of the estimated treatment contrasts for scenarios S1 to S4. Each point on the plots represents a performance measure of an estimate $\psi_{j}$ (with j=T1 to T10 plotted from left to right) provided by either method C (labeled by “△”) or method D (labelled by “∘”). Note that we do not show the relative bias for S1.1 as the true treatment contrasts are all zero, but no absolute bias was larger than 0.02 (see Supplemental Material I Table S2). The numerical values of absolute bias, mean squared error and coverage probability can be found in the Supplemental Material I Tables S2‐S7.

Overall, we find the estimates provided by approaches C and D have similar properties for the scenarios considered, except in S1.2 and S4 where there is discrepancy in the relative bias and coverage probability. Most cases have a small positive bias, a standard finding in logistic regression.^11^ For S1.2, the baseline risk of patients varies across the patterns, and approach D which collapses the odds ratio across such differences shows bias towards the null. If the estimand was the marginal (rather than pattern‐specific) treatment contrasts then approach D would likely have shown little bias and better coverage probability for this scenario (see “Strengths and weaknesses” above).

For S4 where the number of patterns, K, is noticeably larger than the number of possible treatment comparisons (ie, *K =* 200 vs 45 possible treatment comparisons in this scenario) and the baseline risks of all patients are the same, the estimated treatment contrasts from approach D demonstrate smaller bias (and hence smaller mean squared error) than those from approach C. Moreover, method C results in lower coverage probabilities, which is likely due to the large number of covariates in the model. These findings confirm that approach D can provide more accurate estimates for the treatment contrasts when there are many patterns. However, when K is smaller than the number of possible comparisons, as in S3, the estimated treatment contrast from approach D have relatively larger mean squared errors than those from approach C. Nevertheless, they have similar relative bias and the correct coverage.

**FIGURE 2:**
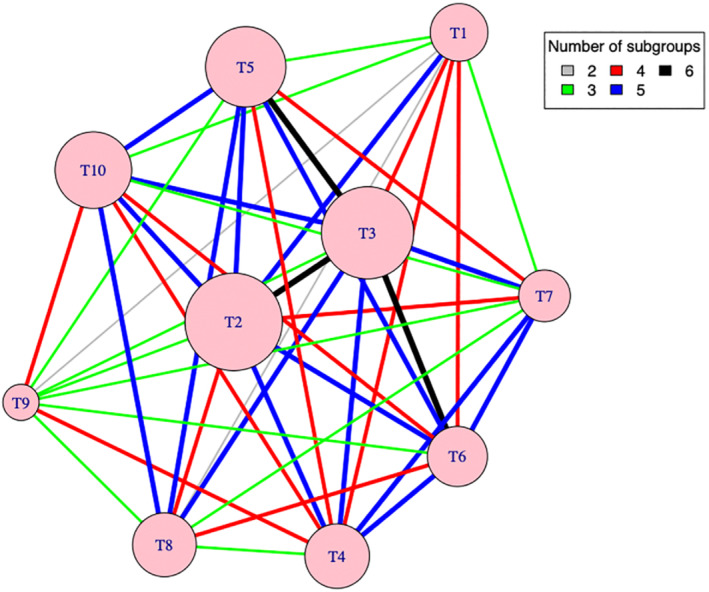
Network map of direct comparisons for scenarios 1, 1.1, and 1.2

Note that one or two of the nine estimated treatment contrasts from either method have a larger magnitude of relative bias and mean squared error for some scenarios. This may be due to small sample bias in logistic regression. For example, Figure 2 shows the network map of direct comparisons for S1, S1.1, and S1.2, where the node size reflects the relative sample size of an arm (contributed by all subgroups) and the thickness of an edge reflects the relative number of patterns contributing to the same direct comparison with color indicating the exact number of subgroups (randomization patterns) contributing to a comparison. For these scenarios arm T9 has the least number of patients among other arms; only two subgroups contribute to the direct comparisons for T9 vs T1 and T8 vs T1, and the amount of information for these comparisons is relatively less (compared to T2 vs T1 and T3 vs T1, for example). This reflects a feature of the PRACTical design: more information is obtained on the most commonly chosen treatment options, which are of most relevance to patients.

When *N* is larger, we find that methods C and D provide estimates that have similar properties for all the scenarios that we have considered so far, except for S1.2 where method D under‐estimates the treatment contrasts to the extent that their mean squared errors are higher than those from method C and that their coverage probabilities are much lower than 0.95. Plots that are similar to those in Figure 1 can be found in the Supplemental Material I Figure Appendix S1 where we have N=10000. This finding confirms that the bias of method C in scenario S4 is a small‐sample phenomenon.

### 5.2 Characteristics of the estimated best treatments

**FIGURE 3:**
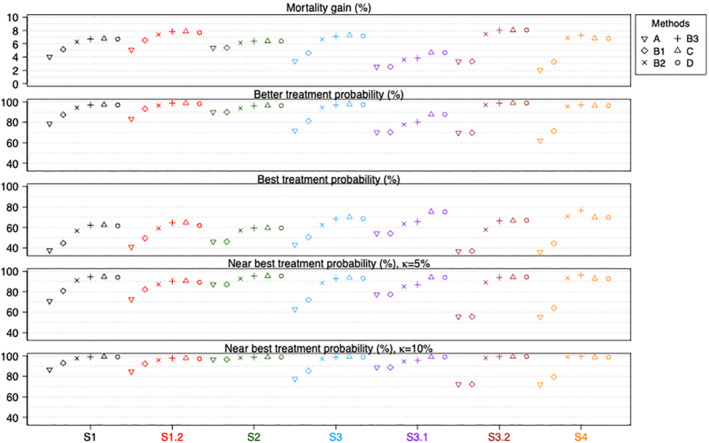
Performance measures of all six methods for scenarios without treatment by pattern interactions. Numerical values and Monte Carlo simulation errors (<0.016 or 1.6% for all cases) are available in Supplemental Material I Tables S8 and S9

We now compare all six analysis approaches in terms of the characteristics of the estimated best treatments for all $S_{k}$. We measure this by the overall mortality gain, better treatment probability, best treatment probability, and near best treatment probability with κ=0.05and0.1 respectively. The κ values were chosen to represent differences that would be of clinical interest. Figure 3 shows these measures for the scenarios that have no treatment by pattern interactions. We omit the results for S1.1 as the true values of all treatment contrasts are zero. The Monte Carlo simulation errors for these measures are small (<0.01) except for methods A and B1 in S4 (due to the smaller number of replications used in the simulation for this particular scenario and because the subgroup sample sizes are small). The numerical values of the measures and Monte Carlo simulation errors can be found in the Supplemental Material I Tables S8 and S9. The number of replications for which a method has a random treatment selection are presented in Supplemental Material I Table S10, where the numbers correspond to the mean, median and maximum value of the frequencies of the patterns in each scenario.

Among the six approaches and for all the considered scenarios without interaction terms, we find that methods B3, C and D perform best with respect to mortality gain, better treatment probability and best treatment probability. The three methods perform similarly in most scenarios except in scenario S3.1 where methods C and D perform better than B3. These results indicate that performing an analysis that makes use of all or nearly all indirect evidence available is a better approach in identifying the best treatment options for patients. Method A uses no indirect evidence, while B1 and B2 use increasing amounts of indirect evidence, and their performance measures for all scenarios without interaction terms are ranked in an order corresponding to the amount of evidence used, with A ranked worst overall.

The fourth and the fifth plots in Figure 3 show how often the estimated best treatments are no more than κ% worse than the most effective treatment in $S_{k}$, with κ=5% and 10%. The rankings of the approaches in terms of this measure are similar in both plots: methods B3, C and D provide the best performance, and method A has the worst performance, for all scenarios.

### 5.3 Scenarios with interactions

**FIGURE 4:**
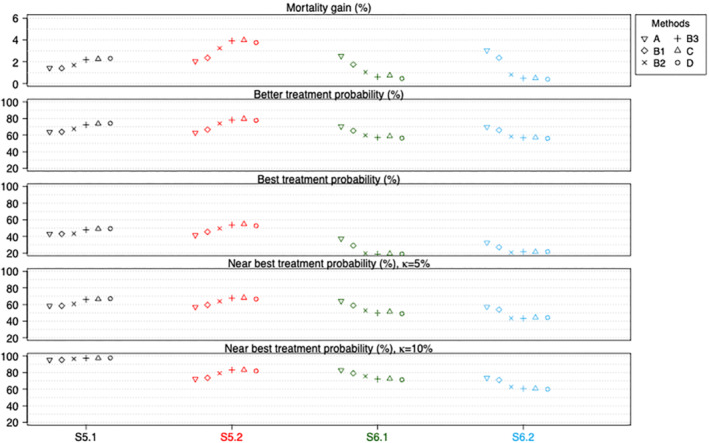
Performance measures of all six methods for scenarios when treatment by pattern interactions exist (quantitative interactions in S5.1 and S5.2; qualitative interactions in S6.1 and S6.2). Numerical values and Monte Carlo simulation errors (<0.0023 or 0.2% for all cases) are available in Supplemental Material I Tables S8 and S9

Figure 4 shows the performance of the six methods when there are quantitative and qualitative treatment by pattern interactions. We find that methods B3, C and D again perform the best when there are quantitative interactions (see S5.1 and S5.2), while method A performs the best when there are qualitative interactions (see S6.1 and S6.2). Methods B1 and B2 perform worse than B3, C and D when there are quantitative interactions but better than B3, C and D when there are qualitative interactions. These findings reflect the bias‐variance trade‐off noted above. These are not surprising because here the data in different patterns are no longer drawn from the same distribution (ie, the location and variability may differ). The corresponding numerical values and Monte Carlo simulation errors are available in the Supplemental Material I Tables S8 and S9.

## 6 DISCUSSION

We have explored six parametric analysis methods for trials that aim to identify the best interventions for patients who have different sets of eligible treatments. The methods fit a model to separate subgroups of patients (method A), fit a model pooling limited information across subgroups (methods B1, B2, and B3), or fit an overall model making use of direct and indirect evidence on treatment comparisons from individual subgroups (methods C and D). The difference between the A and B methods is in the amount of information contributing to the inference about the individual subgroups. (See the Supplemental Material I and Figure S2 for an example that shows the proportion of information contributed to each subgroup analysis.)

For most of the scenarios considered with the parameters of our simulation, we find that methods B2 and B3 outperform A and B1 with respect to identifying the most effective treatment for each respective subgroup, except when qualitative treatment by pattern interactions exist (S6.1 and S6.2), in which case method A performs best. We find that methods C and D provide subgroup rankings that are more precise than those from method B2 and similarly precise to those from method B3 in the scenarios explored. Methods C and D are expected to provide greater precision than the other methods in settings where including large amounts of indirect evidence is appropriate (ie, little interaction). See the Supplemental Material I Figures S3 for an example that shows the direct evidence proportion^12^ for methods C and D. In addition, the overall treatment ranking list produced by methods C and D would be more suitable for practical use, for example, in making intervention policies and guiding individual treatment options, especially when the number of subgroups is large.

Methods A, B1, B2, and B3 can only provide individual estimates for each subgroup for those treatment contrasts included in the corresponding pattern. Applying the methods to the respective subgroups in a study would produce a list of outputs that may have low precision, especially when the sample sizes of the subgroups are small. On the other hand, methods C and D provide an overall finding about the treatment comparisons by including the data of all subgroups and using indirect as well as direct evidence on treatment comparisons. When the total sample size is small and the treatment effects are consistent (ie, no treatment by pattern interactions), we find that the estimates from methods C and D have similar properties with the parameters of our simulation. There are subtle differences when the number of subgroups or patterns is noticeably different to the number of treatment comparisons made in the study.

We note that standard methods for determining sample size (eg, by calculating power to detect a specified difference between treatments) are not applicable to a trial including a network of treatments. A simulation study is required to choose a sample size for a PRACTical design. Supplemental Material I Figure S4 shows an example of sample size evaluation for a trial comparing ten treatments with mortality assumed to range from 10%–30%. A trial including 2000 participants would provide an expected mortality gain of at least 7.8% from choosing the top‐ranked treatment informed by the trial's results rather than choosing a random treatment from each patient's personalised randomization list. It also provides a 99.8% chance that using the top‐ranked treatment would reduce an individual patient's mortality risk in comparison with a randomly chosen treatment (“better treatment probability”), and an 89% chance that the top‐ranked treatment is truly the best treatment (“best treatment probability”). Note that the prevalence rates of the patterns are not known at the design stage. One may estimate these from electronic health records, observational studies or pilot studies. Simulation studies may be conducted with a range of prevalence rates to explore the distribution of the sample size and the performance measures.

Many randomized trials include baseline covariates in order to improve power and precision, and this is also sensible in a PRACTical design. Baseline covariates $x_{i}$ would be included in all the methods described by adding a term $\beta^{\prime }x_{i}$ or $\beta_{\left(k\right)}^{\prime }x_{i}$ to each of the models. The treatment effect parameters $\psi_{j}$ and $\psi_{j\left(k\right)}$ would now be log odds ratios conditional on x, but their ranking would still represent the preferred ranking of the treatments. The performance measures would be modified to averages over all individuals. We expect the results of the simulation study to apply equally to the covariate‐adjusted setting. Sample size calculations could account for covariate adjustment, but this is not usually done.

In settings where comparisons between multiple treatments are of interest but many patients have contra‐indications to certain treatments, and/or there is no obvious single “standard of care”, a PRACTical design allows a randomized trial to be carried out and subsequent treatment decisions to be based on randomized evidence. We have demonstrated that two proposed analytical methods, C and D, perform well with respect to estimation bias and coverage under the scenarios explored. These methods also provide sufficient precision when estimating treatment rankings to produce an overall ranking relating to all patients, which could be used to guide future clinical decisions. The analyses in the illustrations show that the inference is robust to treatment by pattern interactions that are only quantitative. When interactions are qualitative, method C or D are unlikely to be appropriate, since they make less accurate treatment recommendations than the other methods that make inference for the respective subgroups.

Here we focus on treatment rankings as the primary interest of the study. In practice, one can also report other treatment effects, for example, odds ratio, as secondary information. We anticipate that the PRACTical design is most likely to be useful in a Phase IV setting, where treatments have already been licensed for use and their effectiveness has been established, but where information on their comparative effectiveness is lacking. One limitation of our investigation is the limited range of scenarios explored with a fixed sample size in the simulation. In the presence of missing outcome data, the methods we have proposed should remain valid if the data are missing at random. In the presence of intercurrent events such as withdrawal from randomized treatment, our methods also apply if these intercurrent events are handled in the estimand by a treatment policy strategy.^13^ Other handling of intercurrent events requires further investigation.

Future work should develop approaches for estimating performance measures such as mortality gain from real data. Since clinical decisions often depend on multiple factors and not just on efficacy, future work should also investigate performance measures that combine multiple aspects such as clinical efficacy, safety and cost effectiveness in the measures of treatment ranking. Also, we have focused on the context where single treatments are given to patients in a parallel group evaluation. Future research could consider pathways of treatments, such as those in the SMART design where nonresponders are re‐randomized to second‐line treatments.

In conclusion, the PRACTical design and robust analysis approaches can accelerate the evaluation of multiple interventions in settings where randomization between all the interventions may not be possible for all eligible patients. Consideration of treatment rankings can be a new approach to comparing benefits of interventions for scenarios where their efficacies and safety have been proven in other trials.

Sample R scripts are available at https://github.com/kimmaylee215/PRACTical and a simulation study plan that was written before the implementation is available in the Supplemental Material II.

## Supporting information


**Appendix S1.** Supplemental Material I.

**Appendix S2.** Supplemental Material II.

## Data Availability

Data sharing is not applicable to this article as no new data were created or analyzed in this study.
