# Supplementary material for: The Personalised Randomized Controlled Trial: Evaluation of a new trial design
Source: Stat Med. Author manuscript; Available in PMC 2024 Mar 12. (PMC7615735; doi:10.1002/sim.9663)
Supplement: Appendix S1 [file EMS194528-supplement-Appendix_S1.docx]

The Personalised Randomised Controlled Trial (PRACTical): evaluation of a new trial design by Lee et al

This document describes the simulation study plan that was written before implementation.

## Notation for the PRaCTical design

Let a treatment subset or pattern be $S_{k}\subseteq\{T1,\ldots,T10\}$. We use “pattern” to refer to the set of allowable treatments (previously called the “personal randomisation list”) and “subgroup” to refer to the set of patients with this pattern.

Let the probability of pattern $S_{k}$ be $\lambda_{k}$.

Let the intercept (the log odds for a hypothetical neutral treatment) for pattern $S_{k}$ be $\alpha_{k}$.

Patterns used for illustration (not used in simulation):

| $k$ | $S_{k}$ |
| --- | --- |
| 1 | {T1, T2, T3} |
| 2 | { T1, T2, T3, T4} |
| 3 | {T1, T2, T5} |
| 4 | {T1, T3, T6} |
| 5 | {T2, T3, T5} |
| 6 | {T2, T6} |

Let *N* be the total number of patients in the trial.

Let $n_{k}=$*N**$\lambda_{k}$ be the expected sample size of patients who have treatment pattern *k*, $k=1,\ldots,K$. Let $m_{k}$ be the number of eligible treatments in pattern k

Let $\psi_{j}$ be the “effect” of treatment *j* on response probability, $P\left( y_{(i)jk}=1 \right)$ where $y_{(i)jk}$ is the binary response of a patient *i* of pattern *k* receiving treatment *j*, where *j*=1, 2, 3,… corresponding to treatment label T1, T2, T3,…

Patterns remain fixed across all trial replications. Different sets of patterns (i.e. DGM) correspond to different trial examples. Can have equal $\lambda_{k}$ and equal$\alpha_{k}$ for different trial examples. See Table 1.

## Aims of simulation study

1. To compare the performance of different proposed methods of pooled analysis when the treatment effects are the same in all patterns, and to compare with simpler non-pooled methods.
2. To evaluate the performance of one or more methods of pooled analysis when the treatment effects vary across patterns to a plausible extent, and to compare with simpler non-pooled methods.
3. To explore the impact of factors such as number of treatments, number of patterns and risk heterogeneity across patterns on the performance comparisons above.

## Data generating Mechanism

Generate subgroups that are defined by the patterns

1. Randomly assign *N* patients to *K* subgroups using a multinomial distribution with probabilities $\left\{ \lambda_{1},\ldots, \lambda_{K} \right\}$.

Generate treatments

1. For a subgroup *k*, randomly assign $n_{k}$ patients to treatment arms according to pattern $S_{k}$ with equal treatment allocation ratio. E.g. patients of pattern 1 have 1/3 chance of being allocated to each treatment in $S_{1}$.

Generate responses

1. Given the pattern $S_{k}$, simulate responses $y_{\left( i \right)jk}$ using a binomial distribution with $n_{jk}$ and $P_{jk},$ where

$n_{jk}=\sum_{i=1}^{n_{k}} I\left( \text{patient }i\text{ of subgroup }k\text{ receive treatment }j \right)$and

$$P_{jk}=P\left( y_{(i)jk}=1 \right)=\frac{\mathrm{ex}p \left( \alpha_{k}+\psi_{j} \right)}{1+\mathrm{ex}p \left( \alpha_{k}+\psi_{j} \right)} (\text{see below).}$$

Repeat this for all $S_{k}$. Note: binary outcome represents a poor response.

Table 1: parameters of DGM and trial scenarios

| Scenario, S | No. of patterns | Values of $\alpha_{k}$and $\psi_{j}$ | Values of $\lambda_{k}$ |
| --- | --- | --- | --- |
| WITHOUT INTERACTIONS | | | |
| 1 | 8  (from the clinical paper) See page 9 | - Set $\alpha_{k}=\alpha\forall k$  - Choose $\psi_{j}$ such that $P_{jk}$ range from 10% to 30% | - Two patterns each have 20%;  - Six patterns each have 10%  See page 10 |
| 1.1 | Same as S1 | - $\alpha_{k}=\alpha\forall k$ and $\psi_{j}=0 \forall j$ such that $P_{jk}$ = 20% | Same as Scenario 1 |
| 1.2 | Same as S1 | - Choose $\alpha_{k}$ such that   - $P_{jk}$ range (across k) from 5% to 60% when $\psi_{j}=0$, and - pattern $k=1$ with $\lambda_{k}=0.2$ has $P_{jk}=0.2$ when $\psi_{j}=0$.   - In pattern $k=1$, choose $\psi_{j}$ such that $P_{jk}$ range (across j) from 10% to 30%  - Get all $P_{jk}$ from model | Same as Scenario 1 |
| 2 | 3* | Same as S1 | 1/3 |
| 3 | 20*  (5 patterns each with 3 randomly chosen treatments, 5 with 5, 5 with 7, 5 with 9) | Same as S1 | 1/20 |
| 3.1 | 20*  (each with 3 randomly chosen treatments) | Same as S1 | 1/20 |
| 3.2 | 20*  (each with 7 randomly chosen treatments) | Same as S1 | 1/20 |
| 4 | 200*  (UNIQUE patterns (50 patterns each with 3 randomly chosen treatments, 50 with 5, 50 with 7, 50 with 9)) | Same as S1 | 1/200 |
| WITH INTERACTIONS | | | |
| 5.1 | Same as S3 | Choose $\alpha_{k}=$ $\alpha\forall k$ such that $P_{jk}\approx0.20$when $\psi_{j}=0 \forall j$. Then choose $\psi_{j}$ such that $P_{jk}$ range from 10% to 30%; these are the true values.  In each pattern of m treatments, randomly choose $\frac{m_{k}}{2}$ treatments to have $\psi_{j}=0$ while other $\psi_{j}$ remain the same.  To explore the presence of quantitative interactions | 1/20 |
| 5.2 | Same as S3 | Choose $\alpha_{k}$ such that  $P_{jk}$ range (across k) from 5% to 60% when $\psi_{j}=0$.  For the pattern that has $P_{jk}\approx0.20$when $\psi_{j}=0 \forall j$, choose $\psi_{j}$ such that $P_{jk}$ range (across j) from 10% to 30%; these $\psi_{j}$ are the true values for all patterns.  Get all $P_{jk}$ from model.  In each pattern of m treatments, randomly choose $\frac{m_{k}}{2}$ treatments to have $\psi_{j}=0$ while other $\psi_{j}$ remain the same.  To explore the presence of quantitative interactions | 1/20 |
| 6.1 | Same as S3 | Choose $\alpha_{k}=$ $\alpha\forall k$ such that $P_{jk}\approx0.20$when $\psi_{j}=0 \forall j$. Then choose $\psi_{j}$ such that $P_{jk}$ range from 10% to 30%; these are the true values.  In each pattern of m treatments, randomly choose $\frac{m_{k}}{2}$ treatments to negate its $\psi_{j}$ , i.e. if the true value is positive, set it to negative, and vice versa.  To explore the presence of qualitative interactions | 1/20 |
| 6.2 | Same as S3 | Choose $\alpha_{k}$ such that  $P_{jk}$ range (across k) from 5% to 60% when $\psi_{j}=0$.  For the pattern that has $P_{jk}\approx0.20$when $\psi_{j}=0 \forall j$, choose $\psi_{j}$ such that $P_{jk}$ range (across j) from 10% to 30%; these $\psi_{j}$ are the true values for all patterns.  In each pattern of m treatments, randomly choose $\frac{m_{k}}{2}$ treatments to negate its $\psi_{j}$ , i.e. if the true value is positive, set it to negative, and vice versa. To explore the presence of qualitative interactions | 1/20 |

* NB this will be a single randomly drawn set of patterns to be used across all simulated data sets.

Motivation of scenarios:

- S1.2, S5.2 and S6.2: patterns have different baseline risks. Assuming a set of treatment effects for all patterns, we have a setting where a treatment has different response rates when it is administered to patients with different treatment patterns.
- S5.1 and S6.1: patterns have the same baseline mortality rate, say about 20%. Assume a set of treatment effects for all patterns, which consists of effective treatments (that reduce the mortality rate from baseline) and non-effective treatments (that increase mortality rate) respectively.
- To introduce a quantitative interaction term in S5.1 and S5.2, the treatment effect in some patterns are set to zero such that a treatment has no impact on the mortality rate in some patients, but increases or decreases the mortality rate of other patients (depending on the true value of the treatment effect).
- To introduce a qualitative interaction term in S6.1 and S6.2, the treatment effect in some patterns are negated such that an effective treatment becomes ineffective for some patients but not others, and vice versa.
- S5.2 and S6.2: explore the presence of an interaction term as in S5.1 and S6.1 respectively, but with patterns that have different baseline risks.

## Estimands

The estimands are

1. the set of treatment effects $\psi_{j}$
2. the identity of the best treatment in each pattern, with respect to the response (Assume unaffordable treatments and treatments with important toxicities have been excluded from the personal randomization lists.)

## Methods of analysis

How do we draw inferences about the best treatment for a patient in pattern k?

The following analysis approaches are presented in the order of increasing data use (hence increasing assumptions)

Methods A and B do not estimate estimand set 1 (the set of treatment effects $\psi_{j}$) but do estimate estimand set 2 (the best treatment in each pattern). Methods C and D estimate both sets of estimands.

## A: Non-pooled analyses

“Pattern-specific” analysis (Fewest assumptions). Fits to subgroup $k$:

$$logit P\left( y_{jk}=1 \right)=\alpha+\psi_{j}$$

(It will be interesting to compare this analysis with the pooled analyses in A and B in the case of subgroup-treatment interaction. It’s likely to be very inefficient but unbiased.)

## B: partly pooled analyses

Select all data directly relating to comparisons in pattern k. Fit model adjusting for pattern. This is similar to approach D except that this approach will not use the data from the patterns that do not have overlapping treatments.

B1. “Pattern-plus” analysis (simplest extension): use data from other patterns that must include all the treatments of current pattern.

Example: when estimating best treatment

- for pattern 1, use data from pattern 1 and 2 (in illustration on page 1), adjust for the pattern.
- for pattern 2, use data from pattern 2 only as other patterns do not include all the eligible treatments of pattern 2

B2. “Minimal pairwise” analysis (simplest way to use pairwise data): use data from other patterns that include the corresponding pairwise comparison. In particular for pattern k with $m_{k}$ treatments, there are $m_{k}-1$ pairwise comparisons; use a subset of these pairwise comparisons to determine the best treatment. Note that this requires one analysis per pattern, rather than an overall analysis.

Example: for pattern 1, we can

- compare treatment effect of T1 and T2 using data from pattern 1, 2, 3
- compare treatment effect of T1 and T3 using data from pattern 1, 2, 4
- compare treatment effect of T2 and T3 using data from pattern 1, 2, 5.

Various ways to select the best treatment for pattern 1:

1. All pairwise comparisons with first treatment: treatment T1 and T3, and treatment T1 and T2 are compared directly, treatment T2 and T3 are compared indirectly.
2. All pairwise comparisons with previous treatment: compare treatment T1 and T2, and treatment T2 and T3 directly.

We will only consider approach 1 as this is often done in network meta-analysis and is easier to interpret.

B3. “All direct evidence” analysis (uses all direct pairwise evidence): use the data on treatments of the current pattern and data from other patterns that include at least 2 treatments of the current pattern.

Example: when estimating best treatment

- for pattern 1, use data from pattern 1, 2, 3, 4, 5, adjust for the pattern.
- for pattern 2, use data from pattern 1, 2, 3, 4, 5, adjust for the pattern.
- for pattern 3, use data from pattern 1, 2, 3, 5, adjust for the pattern.

Main difference between “All direct evidence” analysis and “Pattern-plus” analysis is that for the pattern with the largest number of eligible treatments, the latter use the data from that pattern only whereas the former would use the data of other patterns that have overlapping eligible treatments.

## C: Network-based approach

Use all individual data to fit the following model

$$logit P\left( y_{(i)jk}=1 \right)=\alpha_{k}+\psi_{j}$$

where $\alpha_{k}$ is the fixed effect of pattern *k*, $\psi_{j}$ is the fixed effect of treatment *j*. Consider treatment as a category variable, we set $\psi_{1}=0$ without loss of generality. The estimated $\psi_{j}$ is interpreted as the effect of treatment *j* relative to treatment one.

This method is likely to be inefficient when there are few (say <5) patients per pattern.

## D: Pairwise trials approach

This method is intended to improve on C when there are few patients per pattern.

Duplicate the data of a pattern *k* ${(m}_{k}-1)$ times such that each duplication is used in a ‘separated’ pairwise comparison (that is feasible for that pattern).

Let *j* denote the treatment received by a patient and $h\subseteq S_{k}, h\neq j$ be the index of other treatments in pattern *k* that the patient has not been allocated to. Each duplicated record must have a different value of $h$. Use all (duplicated) data to fit the following model

$$logit P\left( y_{j(h)}=1 \right)=\alpha_{j,h}+\psi_{j}$$

where $\alpha_{j,h}$ is the fixed effect of making a pairwise comparison between treatment *j* and treatment *h.* We set $\alpha_{j,h}=\alpha_{h,j}$ by randomisation: it represents the expected log odds in a patient eligible for both treatments *j* and *h*, if in fact they received the hypothetical neutral treatment.

For example, all data from pattern 3 are duplicated twice. One duplication of data of a patient who received treatment T1 is used for

1. T1 and T2 comparison, i.e. contribute to the estimation of $\alpha_{T1,T2}+\psi_{T1}$;

and the other duplication for

1. T1 and T5 comparison, i.e. contribute to the estimation of $\alpha_{T1,T5}+\psi_{T1}$.

Likewise, for patients with pattern 3 who received treatment T2 and treatment T5 respectively.

For pattern 2, we duplicate the data thrice. The duplicated data of a patient who received T1 is used for the estimation of $\alpha_{T1,T2}+\psi_{T1}$; $\alpha_{T1,T3}+\psi_{T1}$; and $\alpha_{T1,T4}+\psi_{T1}$. The $\alpha_{T1,T2}$ and $\psi_{T1}$ here are the same parameters as described for pattern 3.

For a trial with ten treatments, there could be a maximum of 45 unique pairwise comparisons. We have less than 45 when some patterns are not overlapping, e.g. the illustration patterns do not have $\alpha_{T1,T7}, \alpha_{T1,T8} , \alpha_{T1,T9} , \alpha_{T1,T10}$ and so on as treatment *T1* vs treatment *T7*, treatment *T1* vs treatment *T8*, treatment *T1* vs treatment *T9*, treatment *T1* vs treatment *T10* comparisons are not available.

The variance-covariance of the estimated parameters needs to be adjusted for within subject ‘correlation’ as we duplicate the individual level data. We consider each individual as a single cluster; we have *N* clusters in our data.

## Performance measures

Let $\Delta=\hat{\boldsymbol{\psi}}-\left( \begin{matrix} \psi_{T2}^{T}-\psi_{T1}^{T} \\ \vdots\\ \psi_{T10}^{T}-\psi_{T1}^{T} \end{matrix} \right)$ be the difference between the estimated model parameters, $\hat{\boldsymbol{\psi}}$, and the true values.

By replicating the trial, we can compute for each treatment contrast,

- bias=$E(\Delta$)
- mean squared error=$E(\Delta^{2})$
- empirical variance of $\hat{\psi}$, i.e. $var(\hat{\psi)}$.
- Coverage: P(*CI* covers the true value)
- Mortality gain: Denote $\hat{j}_{k}=argmin_{j\in S_{k}}\hat{P}\left( k,j \right)$ as the estimated best treatment in pattern $k$ that has the lowest estimated response (mortality) rate $\hat{P}\left( k,j \right)$. Define overall mortality gain of the best treatments as

$$\sum_{k} \hat{\lambda}_{k}G_{k} ,$$

where $\hat{\lambda}_{k}$ is the estimated prevalence rate of pattern *k*, $G_{k}=P\left( k,\hat{j}_{k} \right)-ave_{j}P\left( k,j \right)$ is the mortality gain of the (identified) best treatment in pattern $k$ over the mean of true mortality rates across all other eligible treatments within pattern *k*.

- Better treatment probability: Probability of an improvement in outcome by the (identified) best treatments

$$\sum_{k} \hat{\lambda}_{k}I_{k}$$

where $I_{k}=I\left( P\left( k,\hat{j}_{k} \right)<ave_{j\in S_{k}}P\left( k,j \right) \right)$

- Best treatment probability: Probability that the estimated best treatment is truly the best

$\sum_{k} \lambda_{k}B_{k}$ where $B_{k}=I\left( P\left( k,\hat{j}_{k} \right)={min}_{j\in S_{k}}P\left( k,j \right) \right)$

- Near-best treatment probability: Probability of identifying a near-best treatment defined as one that is no more than $\kappa\%$ worse than the best treatment

$\sum_{k} \lambda_{k}C_{k}$ where $C_{k}=I\left( P\left( k,\hat{j}_{k} \right)\leq{min}_{j\in S_{k}}P\left( k,j \right)+\kappa\right)$ and e.g. $\kappa=0.05$

| Regimen | Patient 1 | Patient 2 | Patient 3 | Patient 4 | Patient 5 | Patient 6 | Patient 7 | Patient 8 |
| --- | --- | --- | --- | --- | --- | --- | --- | --- |
|  | moderate renal impairment | history of myocardial infarction | meropenem MIC ≥64 | VAP/HAP | *Pseudomonas aeruginosa* infection | Known Class B (NDM, IMP, VIM) infection | Presence of 16S-RMT genes** | History of moderate-severe |
|  | (CrCl <40ml/min) |  |  |  |  |  |  | cephalosporin allergy |
| T1: ceftazidime/avibactam | No/maybe† | **Yes** | **Yes** | **Yes** | **Yes** | No | **Yes** | No |
| T2: cefiderocol | **Yes** | **Yes** | **Yes** | **Yes** | **Yes** | **Yes** | **Yes** | No |
| T3: high-dose meropenem* | **Yes** | **Yes** | No | **Yes** | **Yes** | **Yes** | **Yes** | **Yes** |
| T4: polymixin B±zidovudine | No/maybe† | **Yes** | **Yes** | No | **Yes** | **Yes** | **Yes** | **Yes** |
| T5: high-dose meropenem*+ertapenem | **Yes** | **Yes** | No | **Yes** | No | **Yes** | **Yes** | **Yes** |
| T6: high-dose meropenem*+imipenem | No/maybe† | **Yes** | No | **Yes** | **Yes** | **Yes** | **Yes** | **Yes** |
| T7: high-dose meropenem*+polymixin B±zidovudine | No/maybe† | **Yes** | No | No | **Yes** | **Yes** | **Yes** | **Yes** |
| T8: high-dose meropenem*+high-dose tigecyline | **Yes** | No | No | **Yes** | No | **Yes** | **Yes** | **Yes** |
| T9: high-dose tigecyline+polymixin B±zidovudine | No/maybe† | No | **Yes** | No | No | **Yes** | **Yes** | **Yes** |
| T10: high-dose tigecyline+fosfomycin | **Yes** | No | **Yes** | **Yes** | No | **Yes** | **Yes** | **Yes** |
| Pattern | T2, T3, T5, 8, 10 | T1-T7 | T1, T2, T4, T9, T10 | T1-T3,T5,T6,T8,T10 | T1-T4,T6,T7 | T2-T10 | T1-T10 | T3-T10 |
| Number of eligible treatments | 5 | 7 | 5 | 7 | 6 | 9 | 10 | 8 |
| Prevalence (lambda) | 0.2 | 0.2 | 0.1 | 0.1 | 0.1 | 0.1 | 0.1 | 0.1 |
