# Supplementary material for: The Personalised Randomized Controlled Trial: Evaluation of a new trial design
Source: Stat Med. Author manuscript; Available in PMC 2024 Mar 12. (PMC7615735; doi:10.1002/sim.9663)
Supplement: Appendix S2 [file EMS194528-supplement-Appendix_S2.docx]

## Supplemental Material

### I Details of simulation study

This section describes the parameters used in the simulation study. Table S1 describes the parameters used in simulation study. The following are the treatment patterns considered in S1.

Pattern 1: T2, T3, T5, T8, T10

Pattern 2: T1, T2, T3, T4, T5, T6, T7

Pattern 3: T1, T2, T4, T9, T10

Pattern 4: T1, T2, T3, T5, T6, T8, T10

Pattern 5: T1, T2, T3, T4, T6, T7

Pattern 6: T2-T10

Pattern 7: T1-T10

Pattern 8: T3-T10

Patterns 2, 4, and 8 were considered in S2.

**Table S1 Description of scenarios for simulation study**

| Scenario, S | No. of patterns, *k* | Values of $\alpha_{k}$ and $\psi_{j}$ | Values of $\lambda_{k}$ |
| --- | --- | --- | --- |
| WITHOUT INTERACTIONS | | | |
| 1 | 8 | - Set $\alpha_{k}=-1.386294 \forall k$so that $P_{jk}=20\%$ if $\psi_{j}=0$  - Choose $\psi_{j}$ such that $P_{jk}$ range from 10% to 30%, i.e.  $\boldsymbol{\psi=}\left( \psi_{1}, \ldots, \psi_{10} \right)$  $=(-0.811, -0.585, -0.393, -0.223, -0.071, 0.068, 0.197, 0.317, 0.431, 0.539)$ | - $\lambda_{1}=\lambda_{2}=$ 20%;  - $\lambda_{k}=10\%, k=3,...,8$ |
| 1.1 | Same as S1 | - Set $\alpha_{k}=-1.386294 \forall k$ and $\psi_{j}=0 \forall j$ | Same as S1 |
| 1.2 | Same as S1 | - Set $\boldsymbol{\psi}$ as in S1  - Choose $\alpha_{k}$ such that $P_{jk}$ range (across $k$) from 5% to 60% when $\psi_{j}=0$, and | Same as S1 |
| 2 | 3* | Same as S1 | 1/3 |
| 3 | 20*  (5 patterns each with 3 randomly chosen treatments, 5 with 5, 5 with 7, 5 with 9) | Same as S1 | 1/20 |
| 3.1 | 20*  (each with 3 randomly chosen treatments) | Same as S1 | 1/20 |
| 3.2 | 20*  (each with 7 randomly chosen treatments) | Same as S1 | 1/20 |
| 4 | 200*  (UNIQUE patterns: 50 patterns each with 3 randomly chosen treatments, 50 with 5, 50 with 7, 40 with 6, 10 with 9) | Same as S1 | 1/200 |
| WITH INTERACTIONS | | | |
| 5.1 | Same as S3 | Choose $\alpha_{k}$ and $\psi_{j}$ as in S1.  For $k=1,...,20$, randomly choose $\frac{m_{k}}{2}$ treatments in $S_{k}$to have $\psi_{j}=0$ while other $\psi_{j}$ remain the same. | 1/20 |
| 5.2 | Same as S3 | Choose $\alpha_{k}$ and $\psi_{j}$ as in S1.2.  For $k=1,...,20$, randomly choose $\frac{m_{k}}{2}$ treatments in $S_{k}$ to have $\psi_{j}=0$ while other $\psi_{j}$ remain the same. | 1/20 |
| 6.1 | Same as S3 | Choose $\alpha_{k}$ and $\psi_{j}$ as in S1.  For $k=1,...,20$, randomly choose $\frac{m_{k}}{2}$ treatments in $S_{k}$ to negate its $\psi_{j}$ , i.e. if the true value is positive, set it to negative, and vice versa. | 1/20 |
| 6.2 | Same as S3 | Choose $\alpha_{k}$ and $\psi_{j}$ as in S1.2.  For $k=1,...,20$, randomly choose $\frac{m_{k}}{2}$ treatments in $S_{k}$ to negate its $\psi_{j}$ , i.e. if the true value is positive, set it to negative, and vice versa. | 1/20 |

* NB this will be a single randomly drawn set of patterns to be used across all trial replications.

### II Properties of estimates from methods C and D

This section shows the numerical output about the estimated treatment contrasts from methods C and D respectively. Table S2 shows the empirical bias, Table S3 the mean squared error and Table S4 the 95% coverage probability. The corresponding Monte Carlo simulation errors are presented in Table S5-S7. Figure S1 shows the properties of estimated treatment contrasts for scenarios without treatment by pattern interactions when N=10000.

**Table S2 Bias of estimated treatment contrasts.**

|  | *Truth* | S 1 | S 1.1 | S 1.2 | S 2 | S 3 | S 3.1 | S 3.2 | S 4 | S 5.1 | S 5.2 | S 6.1 | S 6.2 |
| --- | --- | --- | --- | --- | --- | --- | --- | --- | --- | --- | --- | --- | --- |
|  |  | Bias of estimated treatment contrast: Method C | | | | | | | | | | | |
| T2 vs T1 | *0.2257* | 0.0339 | 0.0111 | 0.0225 | 0.0144 | 0.0187 | 0.0284 | 0.0150 | 0.0281 | -0.199 | -0.131 | -0.422 | -0.290 |
| T3 vs T1 | *0.4184* | 0.0376 | 0.0065 | 0.0282 | 0.0360 | 0.0314 | 0.0425 | 0.0293 | 0.0514 | -0.208 | -0.168 | -0.470 | -0.354 |
| T4 vs T1 | *0.5878* | 0.0365 | 0.0053 | 0.0286 | 0.0308 | 0.0252 | 0.0437 | 0.0388 | 0.0487 | -0.302 | -0.210 | -0.654 | -0.446 |
| T5 vs T1 | *0.7400* | 0.0461 | 0.0068 | 0.0360 | 0.0409 | 0.0505 | 0.0560 | 0.0476 | 0.0686 | -0.400 | -0.328 | -0.882 | -0.696 |
| T6 vs T1 | *0.8790* | 0.0455 | 0.0036 | 0.0360 | 0.0439 | 0.0486 | 0.0592 | 0.0535 | 0.1041 | -0.529 | -0.440 | -1.146 | -0.933 |
| T7 vs T1 | *1.0076* | 0.0476 | -0.0006 | 0.0396 | 0.0436 | 0.0566 | 0.0727 | 0.0619 | 0.1177 | -0.583 | -0.511 | -1.266 | -1.088 |
| T8 vs T1 | *1.1280* | 0.0537 | 0.0046 | 0.0430 | 0.0461 | 0.0607 | 0.0750 | 0.0642 | 0.1318 | -0.669 | -0.581 | -1.418 | -1.207 |
| T9 vs T1 | *1.2417* | 0.0455 | -0.0131 | 0.0474 | 0.0354 | 0.0658 | 0.0777 | 0.0707 | 0.1487 | -0.730 | -0.650 | -1.548 | -1.347 |
| T10 vs T1 | *1.3499* | 0.0599 | 0.0053 | 0.0464 | 0.0491 | 0.0702 | 0.0801 | 0.0721 | 0.1720 | -0.839 | -0.721 | -1.750 | -1.478 |
|  |  | Bias of estimated treatment contrast: Method D | | | | | | | | | | | |
| T2 vs T1 | *0.2257* | 0.0366 | 0.0113 | -0.0153 | 0.0150 | 0.0190 | 0.0305 | 0.0130 | 0.0084 | -0.129 | -0.098 | -0.300 | -0.207 |
| T3 vs T1 | *0.4184* | 0.0419 | 0.0077 | -0.0245 | 0.0397 | 0.0347 | 0.0414 | 0.0261 | 0.0144 | -0.192 | -0.207 | -0.453 | -0.359 |
| T4 vs T1 | *0.5878* | 0.0398 | 0.0064 | -0.0397 | 0.0346 | 0.0328 | 0.0404 | 0.0338 | -0.0042 | -0.255 | -0.244 | -0.590 | -0.424 |
| T5 vs T1 | *0.7400* | 0.0493 | 0.0079 | -0.0618 | 0.0443 | 0.0543 | 0.0546 | 0.0400 | -0.0001 | -0.353 | -0.357 | -0.812 | -0.652 |
| T6 vs T1 | *0.8790* | 0.0488 | 0.0060 | -0.0905 | 0.0470 | 0.0492 | 0.0552 | 0.0433 | 0.0172 | -0.468 | -0.465 | -1.040 | -0.856 |
| T7 vs T1 | *1.0076* | 0.0502 | 0.0017 | -0.1200 | 0.0474 | 0.0544 | 0.0733 | 0.0493 | 0.0198 | -0.544 | -0.556 | -1.194 | -1.034 |
| T8 vs T1 | *1.1280* | 0.0554 | 0.0043 | -0.0845 | 0.0510 | 0.0586 | 0.0735 | 0.0493 | 0.0210 | -0.603 | -0.606 | -1.300 | -1.126 |
| T9 vs T1 | *1.2417* | 0.0481 | -0.0141 | -0.0980 | 0.0418 | 0.0600 | 0.0743 | 0.0527 | 0.0184 | -0.669 | -0.692 | -1.430 | -1.270 |
| T10 vs T1 | *1.3499* | 0.0602 | 0.0054 | -0.1169 | 0.0541 | 0.0625 | 0.0833 | 0.0527 | 0.0234 | -0.752 | -0.760 | -1.575 | -1.398 |

**Table S3 Mean squared error of estimated treatment contrasts.**

|  | S 1 | S 1.1 | S 1.2 | S 2 | S 3 | S 3.1 | S 3.2 | S 4 | S 5.1 | S 5.2 | S 6.1 | S 6.2 |
| --- | --- | --- | --- | --- | --- | --- | --- | --- | --- | --- | --- | --- |
|  | Mean squared error of estimated treatment contrast: Method C | | | | | | | | | | | |
| T2 vs T1 | 0.2400 | 0.1370 | 0.2057 | 0.2617 | 0.2937 | 0.3621 | 0.3088 | 0.1534 | 0.254 | 0.179 | 0.343 | 0.228 |
| T3 vs T1 | 0.2422 | 0.1424 | 0.1973 | 0.2144 | 0.2469 | 0.3091 | 0.2760 | 0.1414 | 0.220 | 0.167 | 0.357 | 0.247 |
| T4 vs T1 | 0.2511 | 0.1592 | 0.2134 | 0.2425 | 0.2965 | 0.3887 | 0.2532 | 0.1382 | 0.317 | 0.199 | 0.614 | 0.341 |
| T5 vs T1 | 0.2422 | 0.1554 | 0.2067 | 0.2016 | 0.2277 | 0.3423 | 0.2423 | 0.1312 | 0.327 | 0.232 | 0.913 | 0.599 |
| T6 vs T1 | 0.2466 | 0.1688 | 0.2067 | 0.1957 | 0.2351 | 0.3212 | 0.2419 | 0.1354 | 0.461 | 0.335 | 1.468 | 1.000 |
| T7 vs T1 | 0.2538 | 0.1833 | 0.2246 | 0.2233 | 0.2222 | 0.3252 | 0.2275 | 0.1341 | 0.509 | 0.397 | 1.745 | 1.311 |
| T8 vs T1 | 0.2518 | 0.1795 | 0.2109 | 0.2251 | 0.2352 | 0.2825 | 0.2331 | 0.1441 | 0.632 | 0.473 | 2.176 | 1.583 |
| T9 vs T1 | 0.2928 | 0.2394 | 0.2570 | 0.3099 | 0.2083 | 0.2803 | 0.2210 | 0.1383 | 0.688 | 0.550 | 2.527 | 1.931 |
| T10 vs T1 | 0.2300 | 0.1601 | 0.1996 | 0.2182 | 0.2241 | 0.3826 | 0.2280 | 0.1564 | 0.881 | 0.655 | 3.228 | 2.309 |
|  | Mean squared error of estimated treatment contrast: Method D | | | | | | | | | | | |
| T2 vs T1 | 0.2569 | 0.1421 | 0.2014 | 0.2627 | 0.3564 | 0.3633 | 0.3015 | 0.1357 | 0.272 | 0.165 | 0.283 | 0.181 |
| T3 vs T1 | 0.2592 | 0.1480 | 0.1903 | 0.2157 | 0.2933 | 0.3093 | 0.2681 | 0.1267 | 0.248 | 0.176 | 0.365 | 0.248 |
| T4 vs T1 | 0.2688 | 0.1653 | 0.2004 | 0.2451 | 0.3301 | 0.3898 | 0.2456 | 0.1187 | 0.314 | 0.207 | 0.549 | 0.315 |
| T5 vs T1 | 0.2585 | 0.1615 | 0.1993 | 0.2032 | 0.2622 | 0.3435 | 0.2344 | 0.1095 | 0.317 | 0.246 | 0.814 | 0.536 |
| T6 vs T1 | 0.2610 | 0.1722 | 0.2002 | 0.1969 | 0.2780 | 0.3211 | 0.2337 | 0.1077 | 0.433 | 0.352 | 1.259 | 0.860 |
| T7 vs T1 | 0.2688 | 0.1880 | 0.2193 | 0.2258 | 0.2586 | 0.3257 | 0.2192 | 0.1036 | 0.493 | 0.437 | 1.588 | 1.191 |
| T8 vs T1 | 0.2695 | 0.1891 | 0.2105 | 0.2273 | 0.2678 | 0.2830 | 0.2245 | 0.1069 | 0.572 | 0.498 | 1.874 | 1.392 |
| T9 vs T1 | 0.3132 | 0.2540 | 0.2491 | 0.3124 | 0.2433 | 0.2804 | 0.2120 | 0.0943 | 0.635 | 0.599 | 2.202 | 1.727 |
| T10 vs T1 | 0.2454 | 0.1687 | 0.2032 | 0.2207 | 0.2585 | 0.3871 | 0.2181 | 0.1038 | 0.771 | 0.706 | 2.663 | 2.078 |

**Table S4 95% coverage probability of the estimated treatment contrasts.**

|  | S 1 | S 1.1 | S 1.2 | S 2 | S 3 | S 3.1 | S 3.2 | S 4 | S 5.1 | S 5.2 | S 6.1 | S 6.2 |
| --- | --- | --- | --- | --- | --- | --- | --- | --- | --- | --- | --- | --- |
|  | Coverage probability of the estimated treatment contrast: Method C | | | | | | | | | | | |
| T2 vs T1 | 0.9580 | 0.9510 | 0.9521 | 0.9581 | 0.9510 | 0.9515 | 0.9563 | 0.9420 | 0.928 | 0.937 | 0.818 | 0.885 |
| T3 vs T1 | 0.9557 | 0.9507 | 0.9529 | 0.9562 | 0.9542 | 0.9554 | 0.9551 | 0.9400 | 0.921 | 0.922 | 0.752 | 0.820 |
| T4 vs T1 | 0.9570 | 0.9506 | 0.9528 | 0.9564 | 0.9537 | 0.9507 | 0.9542 | 0.9360 | 0.906 | 0.911 | 0.672 | 0.775 |
| T5 vs T1 | 0.9551 | 0.9496 | 0.9542 | 0.9569 | 0.9529 | 0.9526 | 0.9552 | 0.9410 | 0.819 | 0.837 | 0.329 | 0.454 |
| T6 vs T1 | 0.9568 | 0.9508 | 0.9536 | 0.9568 | 0.9532 | 0.9552 | 0.9546 | 0.9440 | 0.739 | 0.770 | 0.156 | 0.253 |
| T7 vs T1 | 0.9586 | 0.9518 | 0.9508 | 0.9547 | 0.9529 | 0.9546 | 0.9559 | 0.9250 | 0.671 | 0.697 | 0.077 | 0.128 |
| T8 vs T1 | 0.9555 | 0.9515 | 0.9516 | 0.9559 | 0.9541 | 0.9549 | 0.9557 | 0.9410 | 0.632 | 0.632 | 0.059 | 0.075 |
| T9 vs T1 | 0.9540 | 0.9516 | 0.9500 | 0.9540 | 0.9550 | 0.9554 | 0.9569 | 0.9210 | 0.513 | 0.536 | 0.013 | 0.026 |
| T10 vs T1 | 0.9571 | 0.9498 | 0.9527 | 0.9562 | 0.9530 | 0.9506 | 0.9567 | 0.9170 | 0.459 | 0.480 | 0.008 | 0.016 |
|  | Coverage probability of the estimated treatment contrast: Method D | | | | | | | | | | | |
| T2 vs T1 | 0.9556 | 0.9533 | 0.9497 | 0.9584 | 0.9472 | 0.9528 | 0.9582 | 0.9510 | 0.941 | 0.946 | 0.893 | 0.917 |
| T3 vs T1 | 0.9537 | 0.9518 | 0.9490 | 0.9578 | 0.9520 | 0.9567 | 0.9572 | 0.9510 | 0.926 | 0.911 | 0.788 | 0.816 |
| T4 vs T1 | 0.9543 | 0.9517 | 0.9490 | 0.9567 | 0.9519 | 0.9518 | 0.9564 | 0.9430 | 0.918 | 0.897 | 0.734 | 0.786 |
| T5 vs T1 | 0.9530 | 0.9507 | 0.9437 | 0.9578 | 0.9512 | 0.9543 | 0.9575 | 0.9560 | 0.854 | 0.812 | 0.444 | 0.488 |
| T6 vs T1 | 0.9549 | 0.9511 | 0.9396 | 0.9577 | 0.9512 | 0.9558 | 0.9573 | 0.9540 | 0.804 | 0.748 | 0.284 | 0.318 |
| T7 vs T1 | 0.9562 | 0.9524 | 0.9317 | 0.9560 | 0.9508 | 0.9552 | 0.9578 | 0.9450 | 0.733 | 0.643 | 0.155 | 0.154 |
| T8 vs T1 | 0.9514 | 0.9500 | 0.9379 | 0.9561 | 0.9506 | 0.9564 | 0.9581 | 0.9630 | 0.710 | 0.600 | 0.132 | 0.109 |
| T9 vs T1 | 0.9517 | 0.9515 | 0.9380 | 0.9545 | 0.9507 | 0.9560 | 0.9580 | 0.9640 | 0.612 | 0.472 | 0.051 | 0.040 |
| T10 vs T1 | 0.9543 | 0.9506 | 0.9296 | 0.9563 | 0.9506 | 0.9514 | 0.9588 | 0.9610 | 0.577 | 0.424 | 0.041 | 0.024 |

**Table S5 Monte Carlo simulation errors: bias of estimated treatment contrasts.**

|  | S 1 | S 1.1 | S 1.2 | S 2 | S 3 | S 3.1 | S 3.2 | S 4 | S 5.1 | S 5.2 | S 6.1 | S 6.2 |
| --- | --- | --- | --- | --- | --- | --- | --- | --- | --- | --- | --- | --- |
|  | Monte Carlo simulation error of the bias: Method C | | | | | | | | | | | |
| T2 vs T1 | 0.0022 | 0.0017 | 0.0020 | 0.0023 | 0.0024 | 0.0027 | 0.0025 | 0.0124 | 0.0021 | 0.0018 | 0.0018 | 0.0017 |
| T3 vs T1 | 0.0022 | 0.0017 | 0.0020 | 0.0021 | 0.0022 | 0.0025 | 0.0023 | 0.0118 | 0.0019 | 0.0017 | 0.0016 | 0.0016 |
| T4 vs T1 | 0.0022 | 0.0018 | 0.0021 | 0.0022 | 0.0024 | 0.0028 | 0.0022 | 0.0117 | 0.0021 | 0.0018 | 0.0019 | 0.0017 |
| T5 vs T1 | 0.0022 | 0.0018 | 0.0020 | 0.0020 | 0.0021 | 0.0026 | 0.0022 | 0.0113 | 0.0018 | 0.0016 | 0.0016 | 0.0015 |
| T6 vs T1 | 0.0022 | 0.0018 | 0.0020 | 0.0020 | 0.0022 | 0.0025 | 0.0022 | 0.0112 | 0.0019 | 0.0017 | 0.0018 | 0.0016 |
| T7 vs T1 | 0.0022 | 0.0019 | 0.0021 | 0.0021 | 0.0021 | 0.0025 | 0.0021 | 0.0110 | 0.0018 | 0.0016 | 0.0017 | 0.0016 |
| T8 vs T1 | 0.0022 | 0.0019 | 0.0020 | 0.0021 | 0.0022 | 0.0024 | 0.0021 | 0.0113 | 0.0019 | 0.0016 | 0.0018 | 0.0016 |
| T9 vs T1 | 0.0024 | 0.0022 | 0.0023 | 0.0025 | 0.0020 | 0.0023 | 0.0021 | 0.0108 | 0.0018 | 0.0016 | 0.0016 | 0.0015 |
| T10 vs T1 | 0.0021 | 0.0018 | 0.0020 | 0.0021 | 0.0021 | 0.0027 | 0.0021 | 0.0113 | 0.0019 | 0.0016 | 0.0018 | 0.0016 |
|  | Monte Carlo simulation error of the bias: Method D | | | | | | | | | | | |
| T2 vs T1 | 0.0023 | 0.0017 | 0.0020 | 0.0023 | 0.0027 | 0.0027 | 0.0025 | 0.0117 | 0.0023 | 0.0018 | 0.0020 | 0.0017 |
| T3 vs T1 | 0.0023 | 0.0017 | 0.0019 | 0.0021 | 0.0024 | 0.0025 | 0.0023 | 0.0113 | 0.0021 | 0.0016 | 0.0018 | 0.0015 |
| T4 vs T1 | 0.0023 | 0.0018 | 0.0020 | 0.0022 | 0.0026 | 0.0028 | 0.0022 | 0.0109 | 0.0022 | 0.0017 | 0.0020 | 0.0016 |
| T5 vs T1 | 0.0023 | 0.0018 | 0.0020 | 0.0020 | 0.0023 | 0.0026 | 0.0022 | 0.0105 | 0.0020 | 0.0015 | 0.0018 | 0.0015 |
| T6 vs T1 | 0.0023 | 0.0019 | 0.0020 | 0.0020 | 0.0023 | 0.0025 | 0.0022 | 0.0104 | 0.0021 | 0.0017 | 0.0019 | 0.0016 |
| T7 vs T1 | 0.0023 | 0.0019 | 0.0020 | 0.0021 | 0.0023 | 0.0025 | 0.0021 | 0.0102 | 0.0020 | 0.0016 | 0.0018 | 0.0016 |
| T8 vs T1 | 0.0023 | 0.0019 | 0.0020 | 0.0021 | 0.0023 | 0.0024 | 0.0021 | 0.0103 | 0.0020 | 0.0016 | 0.0019 | 0.0016 |
| T9 vs T1 | 0.0025 | 0.0023 | 0.0022 | 0.0025 | 0.0022 | 0.0023 | 0.0020 | 0.0097 | 0.0019 | 0.0016 | 0.0018 | 0.0015 |
| T10 vs T1 | 0.0022 | 0.0018 | 0.0019 | 0.0021 | 0.0023 | 0.0028 | 0.0021 | 0.0102 | 0.0020 | 0.0016 | 0.0019 | 0.0016 |

**Table S6 Monte Carlo simulation errors: mean squared error of estimated treatment contrasts.**

|  | S 1 | S 1.1 | S 1.2 | S 2 | S 3 | S 3.1 | S 3.2 | S 4 | S 5.1 | S 5.2 | S 6.1 | S 6.2 |
| --- | --- | --- | --- | --- | --- | --- | --- | --- | --- | --- | --- | --- |
|  | Monte Carlo simulation error of the mean squared error: Method C | | | | | | | | | | | |
| T2 vs T1 | 0.0018 | 0.0009 | 0.0015 | 0.0019 | 0.0021 | 0.0026 | 0.0023 | 0.0079 | 0.0016 | 0.0012 | 0.0019 | 0.0014 |
| T3 vs T1 | 0.0018 | 0.0010 | 0.0014 | 0.0016 | 0.0018 | 0.0022 | 0.0020 | 0.0071 | 0.0014 | 0.0010 | 0.0018 | 0.0013 |
| T4 vs T1 | 0.0019 | 0.0011 | 0.0015 | 0.0018 | 0.0021 | 0.0028 | 0.0019 | 0.0064 | 0.0020 | 0.0012 | 0.0029 | 0.0018 |
| T5 vs T1 | 0.0018 | 0.0010 | 0.0015 | 0.0016 | 0.0016 | 0.0024 | 0.0018 | 0.0066 | 0.0018 | 0.0013 | 0.0030 | 0.0022 |
| T6 vs T1 | 0.0018 | 0.0011 | 0.0015 | 0.0015 | 0.0017 | 0.0023 | 0.0018 | 0.0063 | 0.0023 | 0.0017 | 0.0042 | 0.0031 |
| T7 vs T1 | 0.0019 | 0.0012 | 0.0016 | 0.0017 | 0.0016 | 0.0024 | 0.0018 | 0.0066 | 0.0023 | 0.0019 | 0.0044 | 0.0036 |
| T8 vs T1 | 0.0019 | 0.0012 | 0.0015 | 0.0017 | 0.0017 | 0.0021 | 0.0018 | 0.0075 | 0.0028 | 0.0021 | 0.0053 | 0.0039 |
| T9 vs T1 | 0.0022 | 0.0017 | 0.0018 | 0.0022 | 0.0016 | 0.0021 | 0.0018 | 0.0072 | 0.0027 | 0.0022 | 0.0051 | 0.0042 |
| T10 vs T1 | 0.0018 | 0.0011 | 0.0015 | 0.0017 | 0.0017 | 0.0027 | 0.0018 | 0.0075 | 0.0033 | 0.0025 | 0.0065 | 0.0048 |
|  | Monte Carlo simulation error of the mean squared error: Method D | | | | | | | | | | | |
| T2 vs T1 | 0.0020 | 0.0009 | 0.0015 | 0.0019 | 0.0026 | 0.0026 | 0.0022 | 0.0072 | 0.0018 | 0.0011 | 0.0017 | 0.0011 |
| T3 vs T1 | 0.0020 | 0.0010 | 0.0014 | 0.0016 | 0.0022 | 0.0022 | 0.0020 | 0.0062 | 0.0016 | 0.0011 | 0.0019 | 0.0013 |
| T4 vs T1 | 0.0021 | 0.0011 | 0.0014 | 0.0018 | 0.0024 | 0.0028 | 0.0018 | 0.0057 | 0.0020 | 0.0012 | 0.0028 | 0.0016 |
| T5 vs T1 | 0.0020 | 0.0011 | 0.0014 | 0.0016 | 0.0020 | 0.0025 | 0.0018 | 0.0058 | 0.0018 | 0.0013 | 0.0030 | 0.0021 |
| T6 vs T1 | 0.0020 | 0.0012 | 0.0014 | 0.0016 | 0.0021 | 0.0023 | 0.0018 | 0.0054 | 0.0023 | 0.0017 | 0.0041 | 0.0028 |
| T7 vs T1 | 0.0021 | 0.0013 | 0.0015 | 0.0017 | 0.0020 | 0.0024 | 0.0017 | 0.0052 | 0.0024 | 0.0019 | 0.0044 | 0.0033 |
| T8 vs T1 | 0.0020 | 0.0013 | 0.0014 | 0.0017 | 0.0021 | 0.0022 | 0.0017 | 0.0054 | 0.0027 | 0.0021 | 0.0051 | 0.0036 |
| T9 vs T1 | 0.0023 | 0.0018 | 0.0017 | 0.0022 | 0.0019 | 0.0022 | 0.0017 | 0.0049 | 0.0027 | 0.0022 | 0.0051 | 0.0039 |
| T10 vs T1 | 0.0019 | 0.0011 | 0.0014 | 0.0017 | 0.0020 | 0.0027 | 0.0017 | 0.0051 | 0.0032 | 0.0025 | 0.0062 | 0.0045 |

**Table S7 Monte Carlo simulation errors: coverage probability of estimated treatment contrasts.**

|  | S 1 | S 1.1 | S 1.2 | S 2 | S 3 | S 3.1 | S 3.2 | S 4 | S 5.1 | S 5.2 | S 6.1 | S 6.2 |
| --- | --- | --- | --- | --- | --- | --- | --- | --- | --- | --- | --- | --- |
|  | Monte Carlo simulation error of the coverage probability: Method C | | | | | | | | | | | |
| T2 vs T1 | 0.0009 | 0.0010 | 0.0010 | 0.0009 | 0.0010 | 0.0010 | 0.0009 | 0.0074 | 0.0012 | 0.0011 | 0.0017 | 0.0014 |
| T3 vs T1 | 0.0009 | 0.0010 | 0.0009 | 0.0009 | 0.0009 | 0.0009 | 0.0009 | 0.0075 | 0.0012 | 0.0012 | 0.0019 | 0.0017 |
| T4 vs T1 | 0.0009 | 0.0010 | 0.0009 | 0.0009 | 0.0009 | 0.0010 | 0.0009 | 0.0077 | 0.0013 | 0.0013 | 0.0021 | 0.0019 |
| T5 vs T1 | 0.0009 | 0.0010 | 0.0009 | 0.0009 | 0.0009 | 0.0010 | 0.0009 | 0.0075 | 0.0017 | 0.0017 | 0.0021 | 0.0022 |
| T6 vs T1 | 0.0009 | 0.0010 | 0.0009 | 0.0009 | 0.0009 | 0.0009 | 0.0009 | 0.0073 | 0.0020 | 0.0019 | 0.0016 | 0.0019 |
| T7 vs T1 | 0.0009 | 0.0010 | 0.0010 | 0.0009 | 0.0009 | 0.0009 | 0.0009 | 0.0083 | 0.0021 | 0.0021 | 0.0012 | 0.0015 |
| T8 vs T1 | 0.0009 | 0.0010 | 0.0010 | 0.0009 | 0.0009 | 0.0009 | 0.0009 | 0.0075 | 0.0022 | 0.0022 | 0.0011 | 0.0012 |
| T9 vs T1 | 0.0009 | 0.0010 | 0.0010 | 0.0009 | 0.0009 | 0.0009 | 0.0009 | 0.0085 | 0.0022 | 0.0022 | 0.0005 | 0.0007 |
| T10 vs T1 | 0.0009 | 0.0010 | 0.0009 | 0.0009 | 0.0009 | 0.0010 | 0.0009 | 0.0087 | 0.0022 | 0.0022 | 0.0004 | 0.0006 |
|  | Monte Carlo simulation error of the coverage probability: Method D | | | | | | | | | | | |
| T2 vs T1 | 0.0009 | 0.0009 | 0.0010 | 0.0009 | 0.0010 | 0.0009 | 0.0009 | 0.0068 | 0.0011 | 0.0010 | 0.0014 | 0.0012 |
| T3 vs T1 | 0.0009 | 0.0010 | 0.0010 | 0.0009 | 0.0010 | 0.0009 | 0.0009 | 0.0068 | 0.0012 | 0.0013 | 0.0018 | 0.0017 |
| T4 vs T1 | 0.0009 | 0.0010 | 0.0010 | 0.0009 | 0.0010 | 0.0010 | 0.0009 | 0.0073 | 0.0012 | 0.0014 | 0.0020 | 0.0018 |
| T5 vs T1 | 0.0009 | 0.0010 | 0.0010 | 0.0009 | 0.0010 | 0.0009 | 0.0009 | 0.0065 | 0.0016 | 0.0017 | 0.0022 | 0.0022 |
| T6 vs T1 | 0.0009 | 0.0010 | 0.0011 | 0.0009 | 0.0010 | 0.0009 | 0.0009 | 0.0066 | 0.0018 | 0.0019 | 0.0020 | 0.0021 |
| T7 vs T1 | 0.0009 | 0.0010 | 0.0011 | 0.0009 | 0.0010 | 0.0009 | 0.0009 | 0.0072 | 0.0020 | 0.0021 | 0.0016 | 0.0016 |
| T8 vs T1 | 0.0010 | 0.0010 | 0.0011 | 0.0009 | 0.0010 | 0.0009 | 0.0009 | 0.0060 | 0.0020 | 0.0022 | 0.0015 | 0.0014 |
| T9 vs T1 | 0.0010 | 0.0010 | 0.0011 | 0.0009 | 0.0010 | 0.0009 | 0.0009 | 0.0059 | 0.0022 | 0.0022 | 0.0010 | 0.0009 |
| T10 vs T1 | 0.0009 | 0.0010 | 0.0011 | 0.0009 | 0.0010 | 0.0010 | 0.0009 | 0.0061 | 0.0022 | 0.0022 | 0.0009 | 0.0007 |

**
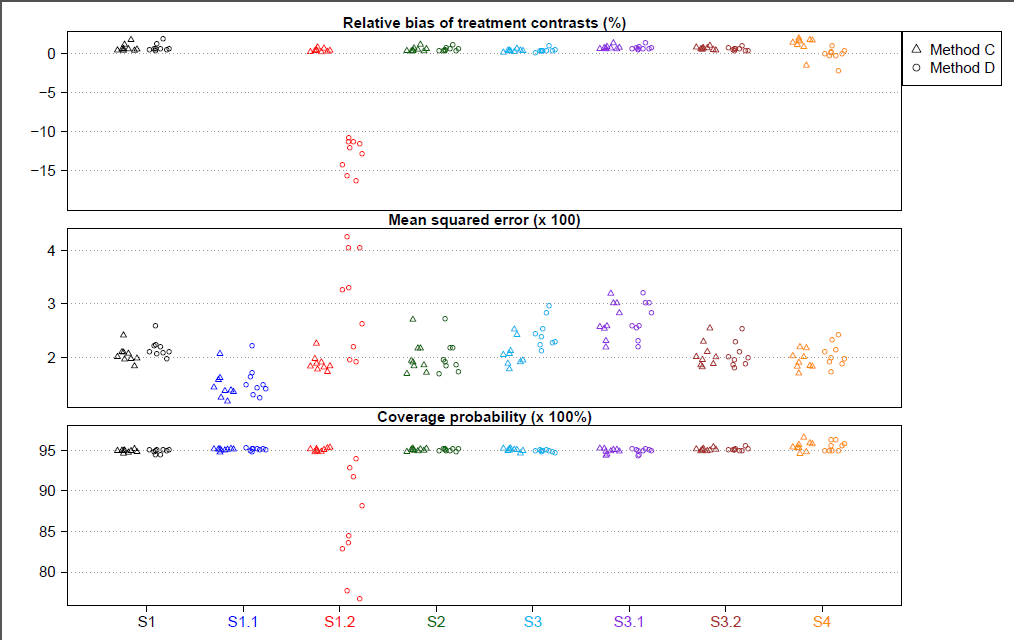
**

**Figure S1: Properties of estimated treatment contrasts for scenarios without treatment by pattern interactions when N=10000. This simulation used 1000 replications for S4 and 10000 replications for all other scenarios.**

### III Numerical values of performance measures

The numerical values of mortality gain, better treatment probability, best treatment probability, and near best treatment probabilities are presented in Table S8. The corresponding Monte Carlo simulation errors are presented in Table S9

**Table S8 Performance measures of all six analysis methods. Scenario S1.1 is excluded as all treatment contrasts are zero. Maximum mortality gains provided by an “oracle” approach for S5.1, 5.2, 6.1 and 6.2 are 0.055, 0.081, 0.073, and 0.092 respectively.**

|  | S1 | S1.2 | S2 | S3 | S3.1 | S3.2 | S4 | S 5.1 | S 5.2 | S 6.1 | S 6.2 |
| --- | --- | --- | --- | --- | --- | --- | --- | --- | --- | --- | --- |
| Mortality Gain | | | | | | | | | | | |
| Method A | 0.0404 | 0.0509 | 0.0539 | 0.0339 | 0.0255 | 0.0333 | 0.0205 | 0.0144 | 0.0206 | 0.0254 | 0.0305 |
| Method B1 | 0.0514 | 0.0652 | 0.0539 | 0.0459 | 0.0255 | 0.0333 | 0.0327 | 0.0141 | 0.0236 | 0.0176 | 0.0236 |
| Method B2 | 0.0628 | 0.0737 | 0.0614 | 0.0666 | 0.0360 | 0.0744 | 0.0688 | 0.0170 | 0.0325 | 0.0105 | 0.0083 |
| Method B3 | 0.0673 | 0.0783 | 0.0638 | 0.0714 | 0.0382 | 0.0802 | 0.0723 | 0.0219 | 0.0392 | 0.0063 | 0.0049 |
| Method C | 0.0676 | 0.0786 | 0.0639 | 0.0725 | 0.0468 | 0.0804 | 0.0680 | 0.0226 | 0.0399 | 0.0075 | 0.0050 |
| Method D | 0.0671 | 0.0768 | 0.0639 | 0.0716 | 0.0468 | 0.0806 | 0.0679 | 0.0231 | 0.0377 | 0.0048 | 0.0041 |
| Better treatment probability | | | | | | | | | | | |
| Method A | 0.7871 | 0.8332 | 0.8994 | 0.7187 | 0.7025 | 0.6969 | 0.6209 | 0.6367 | 0.6287 | 0.7045 | 0.6972 |
| Method B1 | 0.8740 | 0.9323 | 0.8994 | 0.8121 | 0.7025 | 0.6971 | 0.7154 | 0.6385 | 0.6653 | 0.6519 | 0.6593 |
| Method B2 | 0.9435 | 0.9638 | 0.9368 | 0.9440 | 0.7784 | 0.9700 | 0.9555 | 0.6746 | 0.7393 | 0.5966 | 0.5826 |
| Method B3 | 0.9704 | 0.9868 | 0.9622 | 0.9689 | 0.8033 | 0.9878 | 0.9707 | 0.7225 | 0.7815 | 0.5726 | 0.5669 |
| Method C | 0.9718 | 0.9874 | 0.9634 | 0.9761 | 0.8765 | 0.9884 | 0.9621 | 0.7368 | 0.7957 | 0.5857 | 0.5712 |
| Method D | 0.9690 | 0.9819 | 0.9634 | 0.9718 | 0.8764 | 0.9888 | 0.9628 | 0.7411 | 0.7768 | 0.5639 | 0.5596 |
| Best treatment probability | | | | | | | | | | | |
| Method A | 0.3767 | 0.4096 | 0.4617 | 0.4320 | 0.5420 | 0.3687 | 0.3606 | 0.4308 | 0.4155 | 0.3745 | 0.3256 |
| Method B1 | 0.4465 | 0.4953 | 0.4617 | 0.5060 | 0.5419 | 0.3687 | 0.4436 | 0.4304 | 0.4551 | 0.2899 | 0.2704 |
| Method B2 | 0.5676 | 0.5926 | 0.5713 | 0.6246 | 0.6349 | 0.5793 | 0.7086 | 0.4342 | 0.4964 | 0.1955 | 0.2063 |
| Method B3 | 0.6204 | 0.6449 | 0.5945 | 0.6852 | 0.6557 | 0.6633 | 0.7665 | 0.4809 | 0.5381 | 0.1875 | 0.2152 |
| Method C | 0.6240 | 0.6483 | 0.5956 | 0.7008 | 0.7535 | 0.6669 | 0.6990 | 0.4918 | 0.5485 | 0.1918 | 0.2135 |
| Method D | 0.6162 | 0.6197 | 0.5950 | 0.6859 | 0.7529 | 0.6694 | 0.6983 | 0.4942 | 0.5279 | 0.1890 | 0.2169 |
| Near best treatment probability, $\kappa=5$% | | | | | | | | | | | |
| Method A | 0.7072 | 0.7255 | 0.8711 | 0.6291 | 0.7735 | 0.5568 | 0.5546 | 0.5842 | 0.5709 | 0.6419 | 0.5725 |
| Method B1 | 0.8081 | 0.8217 | 0.8710 | 0.7209 | 0.7735 | 0.5568 | 0.6418 | 0.5843 | 0.5948 | 0.5890 | 0.5392 |
| Method B2 | 0.9101 | 0.8719 | 0.9272 | 0.8854 | 0.8499 | 0.8903 | 0.9351 | 0.6068 | 0.6377 | 0.5278 | 0.4349 |
| Method B3 | 0.9433 | 0.9037 | 0.9526 | 0.9287 | 0.8688 | 0.9396 | 0.9625 | 0.6585 | 0.6767 | 0.4975 | 0.4330 |
| Method C | 0.9451 | 0.9056 | 0.9537 | 0.9376 | 0.9400 | 0.9413 | 0.9276 | 0.6646 | 0.6799 | 0.5135 | 0.4432 |
| Method D | 0.9405 | 0.8914 | 0.9535 | 0.9317 | 0.9400 | 0.9428 | 0.9273 | 0.6701 | 0.6652 | 0.4901 | 0.4431 |
| Near best treatment probability, $\kappa=10$% | | | | | | | | | | | |
| Method A | 0.8667 | 0.8468 | 0.9653 | 0.7752 | 0.8881 | 0.7228 | 0.7218 | 0.9523 | 0.7237 | 0.8289 | 0.7368 |
| Method B1 | 0.9299 | 0.9240 | 0.9654 | 0.8536 | 0.8879 | 0.7229 | 0.7955 | 0.9521 | 0.7366 | 0.7912 | 0.7107 |
| Method B2 | 0.9767 | 0.9589 | 0.9819 | 0.9714 | 0.9460 | 0.9797 | 0.9916 | 0.9649 | 0.7909 | 0.7550 | 0.6276 |
| Method B3 | 0.9910 | 0.9784 | 0.9887 | 0.9880 | 0.9562 | 0.9928 | 0.9974 | 0.9743 | 0.8304 | 0.7203 | 0.6074 |
| Method C | 0.9916 | 0.9789 | 0.9889 | 0.9919 | 0.9899 | 0.9932 | 0.9868 | 0.9740 | 0.8324 | 0.7256 | 0.6080 |
| Method D | 0.9905 | 0.9725 | 0.9888 | 0.9890 | 0.9900 | 0.9936 | 0.9870 | 0.9766 | 0.8192 | 0.7143 | 0.5998 |

**Table S9 Monte Carlo simulation error for the performance measures. Scenario S1.1 is excluded as all treatment contrasts are zero. A magnitude of 0.01 for probabilities measures indicate an error of 1%.**

|  | S1 | S1.2 | S2 | S3 | S3.1 | S3.2 | S4 | S 5.1 | S 5.2 | S 6.1 | S 6.2 |
| --- | --- | --- | --- | --- | --- | --- | --- | --- | --- | --- | --- |
| Monte Carlo simulation error for mortality gain | | | | | | | | | | | |
| Method A | 7.14E-05 | 7.79E-05 | 7.94E-05 | 5.80E-05 | 4.60E-05 | 6.16E-05 | 1.37E-04 | 3.86E-05 | 5.59E-05 | 5.40E-05 | 6.54E-05 |
| Method B1 | 6.77E-05 | 7.92E-05 | 7.93E-05 | 5.57E-05 | 4.60E-05 | 6.16E-05 | 2.34E-04 | 4.25E-05 | 6.63E-05 | 5.31E-05 | 6.78E-05 |
| Method B2 | 7.50E-05 | 7.85E-05 | 7.73E-05 | 6.28E-05 | 4.31E-05 | 6.77E-05 | 2.57E-04 | 6.39E-05 | 9.39E-05 | 4.78E-05 | 5.49E-05 |
| Method B3 | 6.04E-05 | 6.16E-05 | 6.53E-05 | 5.03E-05 | 4.00E-05 | 5.63E-05 | 2.04E-04 | 6.27E-05 | 8.63E-05 | 4.09E-05 | 4.94E-05 |
| Method C | 6.23E-05 | 6.40E-05 | 6.57E-05 | 5.25E-05 | 3.14E-05 | 5.90E-05 | 1.81E-04 | 6.76E-05 | 9.11E-05 | 3.65E-05 | 4.24E-05 |
| Method D | 6.42E-05 | 7.00E-05 | 6.57E-05 | 5.51E-05 | 3.13E-05 | 5.80E-05 | 1.79E-04 | 6.73E-05 | 9.40E-05 | 4.00E-05 | 4.41E-05 |
| Monte Carlo simulation error for better treatment probability | | | | | | | | | | | |
| Method A | 1.83E-03 | 1.67E-03 | 1.35E-03 | 2.01E-03 | 2.04E-03 | 2.06E-03 | 1.53E-02 | 2.15E-03 | 2.16E-03 | 2.04E-03 | 2.05E-03 |
| Method B1 | 1.48E-03 | 1.12E-03 | 1.35E-03 | 1.75E-03 | 2.04E-03 | 2.06E-03 | 1.43E-02 | 2.15E-03 | 2.11E-03 | 2.13E-03 | 2.12E-03 |
| Method B2 | 1.03E-03 | 8.36E-04 | 1.09E-03 | 1.03E-03 | 1.86E-03 | 7.63E-04 | 6.52E-03 | 2.10E-03 | 1.96E-03 | 2.19E-03 | 2.21E-03 |
| Method B3 | 7.57E-04 | 5.10E-04 | 8.53E-04 | 7.76E-04 | 1.78E-03 | 4.90E-04 | 5.34E-03 | 2.00E-03 | 1.85E-03 | 2.21E-03 | 2.22E-03 |
| Method C | 7.40E-04 | 4.98E-04 | 8.40E-04 | 6.82E-04 | 1.47E-03 | 4.80E-04 | 6.04E-03 | 1.97E-03 | 1.80E-03 | 2.20E-03 | 2.21E-03 |
| Method D | 7.75E-04 | 5.96E-04 | 8.40E-04 | 7.40E-04 | 1.47E-03 | 4.70E-04 | 5.99E-03 | 1.96E-03 | 1.86E-03 | 2.22E-03 | 2.22E-03 |
| Monte Carlo simulation error for best treatment probability | | | | | | | | | | | |
| Method A | 2.17E-03 | 2.20E-03 | 2.23E-03 | 2.22E-03 | 2.23E-03 | 2.16E-03 | 1.52E-02 | 2.21E-03 | 2.20E-03 | 2.16E-03 | 2.10E-03 |
| Method B1 | 2.22E-03 | 2.24E-03 | 2.23E-03 | 2.24E-03 | 2.23E-03 | 2.16E-03 | 1.57E-02 | 2.21E-03 | 2.23E-03 | 2.03E-03 | 1.99E-03 |
| Method B2 | 2.22E-03 | 2.20E-03 | 2.21E-03 | 2.17E-03 | 2.15E-03 | 2.21E-03 | 1.44E-02 | 2.22E-03 | 2.24E-03 | 1.77E-03 | 1.81E-03 |
| Method B3 | 2.17E-03 | 2.14E-03 | 2.20E-03 | 2.08E-03 | 2.12E-03 | 2.11E-03 | 1.34E-02 | 2.23E-03 | 2.23E-03 | 1.75E-03 | 1.84E-03 |
| Method C | 2.17E-03 | 2.14E-03 | 2.19E-03 | 2.05E-03 | 1.93E-03 | 2.11E-03 | 1.45E-02 | 2.24E-03 | 2.23E-03 | 1.76E-03 | 1.83E-03 |
| Method D | 2.17E-03 | 2.17E-03 | 2.20E-03 | 2.08E-03 | 1.93E-03 | 2.10E-03 | 1.45E-02 | 2.24E-03 | 2.23E-03 | 1.75E-03 | 1.84E-03 |
| Monte Carlo simulation error for near best treatment probability | | | | | | | | | | | |
| Method A | 2.04E-03 | 2.00E-03 | 1.50E-03 | 2.16E-03 | 1.87E-03 | 2.22E-03 | 1.57E-02 | 2.20E-03 | 2.21E-03 | 2.14E-03 | 2.21E-03 |
| Method B1 | 1.76E-03 | 1.71E-03 | 1.50E-03 | 2.01E-03 | 1.87E-03 | 2.22E-03 | 1.52E-02 | 2.20E-03 | 2.20E-03 | 2.20E-03 | 2.23E-03 |
| Method B2 | 1.28E-03 | 1.49E-03 | 1.16E-03 | 1.42E-03 | 1.60E-03 | 1.40E-03 | 7.79E-03 | 2.18E-03 | 2.15E-03 | 2.23E-03 | 2.22E-03 |
| Method B3 | 1.03E-03 | 1.32E-03 | 9.50E-04 | 1.15E-03 | 1.51E-03 | 1.07E-03 | 6.00E-03 | 2.12E-03 | 2.09E-03 | 2.24E-03 | 2.22E-03 |
| Method C | 1.02E-03 | 1.31E-03 | 9.40E-04 | 1.08E-03 | 1.06E-03 | 1.05E-03 | 8.20E-03 | 2.11E-03 | 2.09E-03 | 2.24E-03 | 2.22E-03 |
| Method D | 1.06E-03 | 1.39E-03 | 9.41E-04 | 1.13E-03 | 1.06E-03 | 1.04E-03 | 8.21E-03 | 2.10E-03 | 2.11E-03 | 2.24E-03 | 2.22E-03 |
| Monte Carlo simulation error for near best treatment probability | | | | | | | | | | | |
| Method A | 1.52E-03 | 1.61E-03 | 8.18E-04 | 1.87E-03 | 1.41E-03 | 2.00E-03 | 1.42E-02 | 9.53E-04 | 2.00E-03 | 1.68E-03 | 1.97E-03 |
| Method B1 | 1.14E-03 | 1.19E-03 | 8.18E-04 | 1.58E-03 | 1.41E-03 | 2.00E-03 | 1.28E-02 | 9.55E-04 | 1.97E-03 | 1.82E-03 | 2.03E-03 |
| Method B2 | 6.75E-04 | 8.88E-04 | 5.97E-04 | 7.45E-04 | 1.01E-03 | 6.31E-04 | 2.88E-03 | 8.23E-04 | 1.82E-03 | 1.92E-03 | 2.16E-03 |
| Method B3 | 4.22E-04 | 6.51E-04 | 4.73E-04 | 4.87E-04 | 9.15E-04 | 3.79E-04 | 1.62E-03 | 7.08E-04 | 1.68E-03 | 2.01E-03 | 2.18E-03 |
| Method C | 4.07E-04 | 6.43E-04 | 4.69E-04 | 4.00E-04 | 4.47E-04 | 3.68E-04 | 3.60E-03 | 7.11E-04 | 1.67E-03 | 2.00E-03 | 2.18E-03 |
| Method D | 4.34E-04 | 7.32E-04 | 4.70E-04 | 4.67E-04 | 4.46E-04 | 3.57E-04 | 3.58E-03 | 6.77E-04 | 1.72E-03 | 2.02E-03 | 2.19E-03 |

**Table S10 The number of replications for which a method has a random treatment selection when the analysis model fails to converge. The numbers correspond to the mean, median and maximum value of the frequencies across the patterns in each scenario. Number of replications are 50000 for all scenarios except S4 that has 1000 replications.**

| Scenario | Method A | Method B1 | Method B2 | Method B3 | Method C | Method D |
| --- | --- | --- | --- | --- | --- | --- |
| S1 | 5190, 5720, 7487 | 2049, 1731, 5726 | 8, 13, 13 | 8, 13, 13 | 5, 0, 13 | 5, 0, 13 |
| S1.1 | 1484, 1596, 2542 | 364, 78, 1947 | 0, 0, 0 | 0, 0, 0 | 0, 0, 0 | 0, 0, 0 |
| S1.2 | 2748, 1378, 9931 | 256, 90, 971 | 0, 0, 0 | 0, 0, 0 | 0, 0, 0 | 0, 0, 0 |
| S2 | 342, 382, 575 | 342, 382, 575 | 3, 4, 4 | 3, 4, 4 | 1, 0, 4 | 1, 0, 4 |
| S3.1 | 5224, 5238, 10340 | 2228, 1316, 7198 | 5, 1, 23 | 5, 1, 23 | 0, 0, 1 | 0, 0, 1 |
| S3.2 | 5231, 5375, 14050 | 5231, 5375, 14050 | 714, 116, 4186 | 732, 116, 4295 | 14, 18, 18 | 14, 18, 18 |
| S3.3 | 4990, 4948, 9843 | 4990, 4948, 9843 | 6, 8, 8 | 6, 8, 8 | 3, 0, 8 | 3, 0, 8 |
| S4 | 164, 122, 427 | 91, 86, 252 | 0, 0, 0 | 0, 0, 0 | 0, 0, 0 | 0, 0, 0 |
| S5.1 | 3777, 3493, 10920 | 1352, 752, 6860 | 0, 0, 1 | 0, 0, 1 | 0, 0, 0 | 0, 0, 0 |
| S5.2 | 5915, 5131, 14166 | 1200, 4, 5369 | 0, 0, 0 | 0, 0, 0 | 0, 0, 0 | 0, 0, 0 |
| S6.1 | 2984, 2486, 10286 | 928, 180, 6177 | 0, 0, 0 | 0, 0, 0 | 0, 0, 0 | 0, 0, 0 |
| S6.2 | 5320, 5044, 13791 | 1504, 0, 5535 | 0, 0, 0 | 0, 0, 0 | 0, 0, 0 | 0, 0, 0 |

### IV Proportion of information contributed to each subgroup analysis

Figure S2 shows how patients from different patterns are distributed across the two arms of a direct comparison for a single trial replication of S1 with N=10000, where the direct comparisons involving A as the reference treatment are indicated on the x-axis. The numbers in the bars are computed as follows:

$$\frac{number of patients in pattern k who receive either treatment X or A}{total number of patients in arms X and A}, X=B,C,...,I$$

For a single trial replication, Figure S2 shows there are fewer patients contributing to the contrast between treatments H and A, and only two subgroups provide direct comparison for this contrast. We also see that patterns 1, 6 and 8 are not involved in any direct comparisons that include treatment A, since treatment A is not an eligible treatment for the patients in these patterns. For the analysis, method A would use the data of the same colour code for making inference about the pattern. On the other hand, methods B1, B2 and B3 would pool the relevant data from other subgroups when making inference for a pattern. All of these methods would consider a different reference treatment for patterns not including treatment A.

**Figure S2 Aggregated proportion for each treatment contrast, with colour code indicating the patterns in a single trial replication of S1 with N=10000.**

### V Direct evidence of methods C and D

For methods C and D, the amount of information used in the analysis is similar to the idea of direct and indirect evidence that are often considered in the context of network-meta analysis. Figure S3 shows the direct evidence proportion for the same trial replication considered in the above section. These direct evidence proportions are computed using the netsplit function on R, with back-calculation method^12^, where the results of each pattern from method A are combined using the standard network-meta analysis approach.

**Figure S3 Direct evidence proportion computed using back-calculation methods.**

### VI Example of sample size for PRACTical design with data generating mechanism of scenario S1

**Figure S4 Performance measures of method C for different sample sizes. To choose a sample size for a planned trial, the trial team needs to examine the performance measures reflecting information provided by particular sample sizes.**
